# Extracellular matrix scaffold and hydrogel derived from decellularized and delipidized human pancreas

**DOI:** 10.1038/s41598-018-28857-1

**Published:** 2018-07-11

**Authors:** Sara Dutton Sackett, Daniel M. Tremmel, Fengfei Ma, Austin K. Feeney, Rachel M. Maguire, Matthew E. Brown, Ying Zhou, Xiang Li, Cori O’Brien, Lingjun Li, William J. Burlingham, Jon S. Odorico

**Affiliations:** 1 Division of Transplantation, Department of Surgery, University of Wisconsin-Madison School of Medicine and Public Health, Madison, Wisconsin 53705 USA; 2 School of Pharmacy, University of Wisconsin, Madison, Wisconsin 53705 USA; 3 Department of Chemistry, University of Wisconsin, Madison, Wisconsin 53705 USA

## Abstract

Extracellular matrix (ECM) plays an important developmental role by regulating cell behaviour through structural and biochemical stimulation. Tissue-specific ECM, attained through decellularization, has been proposed in several strategies for tissue and organ replacement. Decellularization of animal pancreata has been reported, but the same methods applied to human pancreas are less effective due to higher lipid content. Moreover, ECM-derived hydrogels can be obtained from many decellularized tissues, but methods have not been reported to obtain human pancreas-derived hydrogel. Using novel decellularization methods with human pancreas we produced an acellular, 3D biological scaffold (hP-ECM) and hydrogel (hP-HG) amenable to tissue culture, transplantation and proteomic applications. The inclusion of a homogenization step in the decellularization protocol significantly improved lipid removal and gelation capability of the resulting ECM, which was capable of gelation at 37 °C *in vitro* and *in vivo*, and is cytocompatible with a variety of cell types and islet-like tissues *in vitro*. Overall, this study demonstrates the characterisation of a novel protocol for the decellularization and delipidization of human pancreatic tissue for the production of acellular ECM and ECM hydrogel suitable for cell culture and transplantation applications. We also report a list of 120 proteins present within the human pancreatic matrisome.

## Introduction

Biological scaffolds derived from extracellular matrix (ECM) have been widely utilised in regenerative medicine^1–3^. The ECM is an essential non-cellular component of the tissue microenvironment, comprised of a network of macromolecules including polysaccharide glycosaminoglycans (GAGs) and proteins such as collagens, laminins, and fibronectin^2,4^. In addition to providing structural support to cells, ECM can guide cell migration, proliferation, differentiation and maturation throughout development as well as influence cell function and differentiation *in vitro*^5–9^. Scaffolds designed for tissue engineering ideally include these ECM ligands to mimic cues within the native microenvironment. To obtain natural ECM, various organs and tissues have been decellularized, via a number of techniques utilizing chemical, enzymatic, or mechanical disruption. Decellularization protocols can be designed to address factors such as tissue density, cellularity and lipid content^1,10^. This is especially important for tissues containing fats such as bone^11^, adipose^12^, and brain^13^, for which decellularization has been combined with a delipidization step, but this process has not been reported for the human pancreas.

Worldwide it is estimated that 387 million people have diabetes and this number is expected to increase by 53% to 592 million by 2035^14,15^. Despite continuing advances in insulin delivery technology and recombinant insulins, diabetes and its complications still claim the lives of millions of people, largely due to imperfect long-standing glycaemic control resulting in end-organ complications. On the other hand, beta cell replacement therapies including whole vascularized pancreas transplantation or the transplantation of isolated islets of Langerhans are able to fully restore normoglycemia, achieve insulin-independence and can delay end-organ complications^16–18^. However, these therapies suffer from several key limitations: the shortage of organs, inconsistent quality of donor organs, and the need for life-long immunosuppression to prevent allograft rejection^19,20^. Many envision a tissue engineering solution, via merging a beta cell source, such as stem cell-derived beta cells, with other cellular and matrix components including natural ECM or biomimetic scaffolds, to address this pressing clinical problem.

The composition and organisation of ECM varies from tissue to tissue^21,22^, however the basic function of all ECM is to provide support for the tissue and ligands for cellular attachment. The relationship between cells and ECM in developing organs is a complex and continuous interplay; cells synthesize and deposit macromolecules that influence the growth and remodelling of the organ, and the deposited ECM supports cell survival, function and organisation throughout life. For example, data show that cell-matrix interactions are important for mature beta cells to remain functional and avoid apoptosis^23^, as well as for maintaining a functional beta cell mass (reviewed in^24^). The ECM in the periphery of the islet has been reported to contain collagen I, III, IV, V and VI, as well as laminin and fibronectin^24^. Convincing data show that islets are often stripped of a large amount of their ECM and dense vascular networks during the isolation process^25–28^. Moreover, islets which retain some of their ECM following isolation exhibit reduced rates of apoptosis and maintain significantly better functional insulin responses than do more aggressively purified islets^29^. Tissue specific ECM sources would provide cellular environments that closely recapitulate the *in vivo* milieu by harnessing the distinctive properties and therefore provide a potential platform for tissue engineering that can specifically enhance the cells ability to function more similarly to that of the original tissue.

Surprisingly, many human pancreata are discarded after recovery from cadaveric donors, which represents a lost precious resource. Presently in the US, only ~17% of donor pancreata are recovered for transplantation^30–32^ and ~25% of those recovered with intent to transplant are discarded^32^. While there are multiple reasons for not recovering pancreata or not transplanting after recovery, numerous organs are available for research through organ procurement organisations. We suggest that these pancreata be utilised to study the composition of the human pancreatic ECM, and potentially be used for bioengineering and regenerative medicine purposes. Donor selection is very stringent for pancreas transplant, and as a result many are declined based on medical history, fibrosis, fat deposition and other conservative practises^33^ even though the pancreas is healthy and functional. These pancreata could be decellularized, processed to construct biomaterials, such as hydrogels, and used for tissue engineering applications rather than be discarded. Hydrogels are versatile materials possessing a number of potential applications, including 2D and 3D scaffolds for cell culture, in which cells can be plated on or embedded within the gel. However, the high lipid content of non-transplantable human pancreata has posed a barrier to achieving adequate decellularization and hydrogel formation using methods that are typically sufficient for decellularizing lean organs and tissues.

The objectives of this study were to develop methods for efficient decellularization and delipidization of human pancreata and to produce a hydrogel amenable to tissue culture and transplantation applications. We describe a novel method to effectively decellularize and remove lipids from human pancreata and have characterised the composition and structure of the acellular human pancreatic ECM (hP-ECM). Furthermore, we demonstrate its ability to form a hydrogel (hP-HG) and use as a viable cell culture platform which sustains cell growth and viability in *in vitro* and *in vivo* environments.

## Results

### Human pancreas decellularization and hydrogel formation

**Figure 1:**
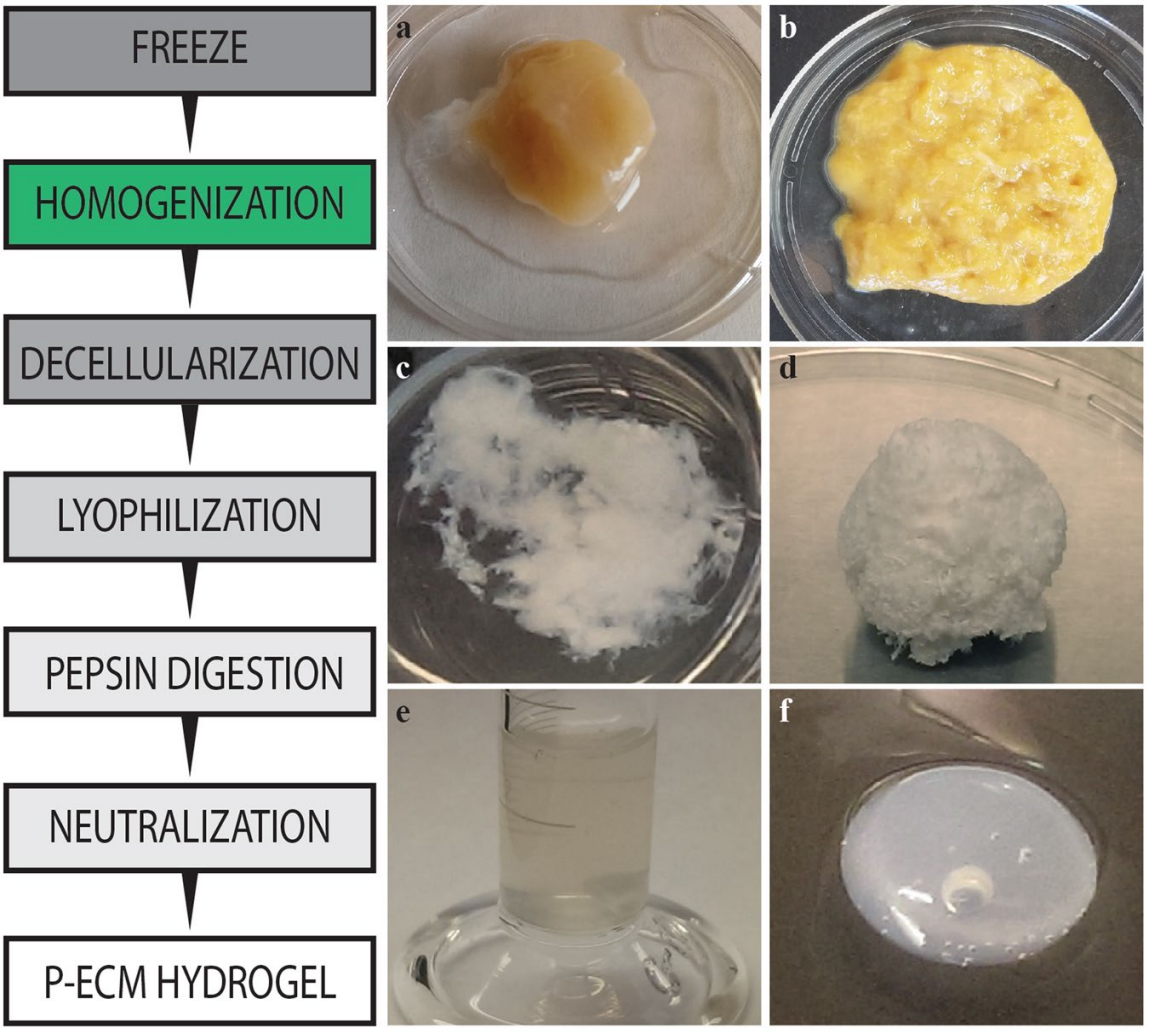
Schematic representation of the preparation of a decellularized ECM hydrogel from pieces of human pancreatic parenchyma. Native tissue is frozen and thawed (**a**), then homogenized (**b**) and decellularized with deoxycholate (**c**). The resulting acellular matrix is lyophilized (**d**) and digested in pepsin/HCl to create a solubilized pancreatic matrix which is liquid at 4 °C (**e**). Following neutralization and warming to 37 °C the hydrogel material self-assembles into a fibrous 3D gel (**f**).

**Figure 2:**
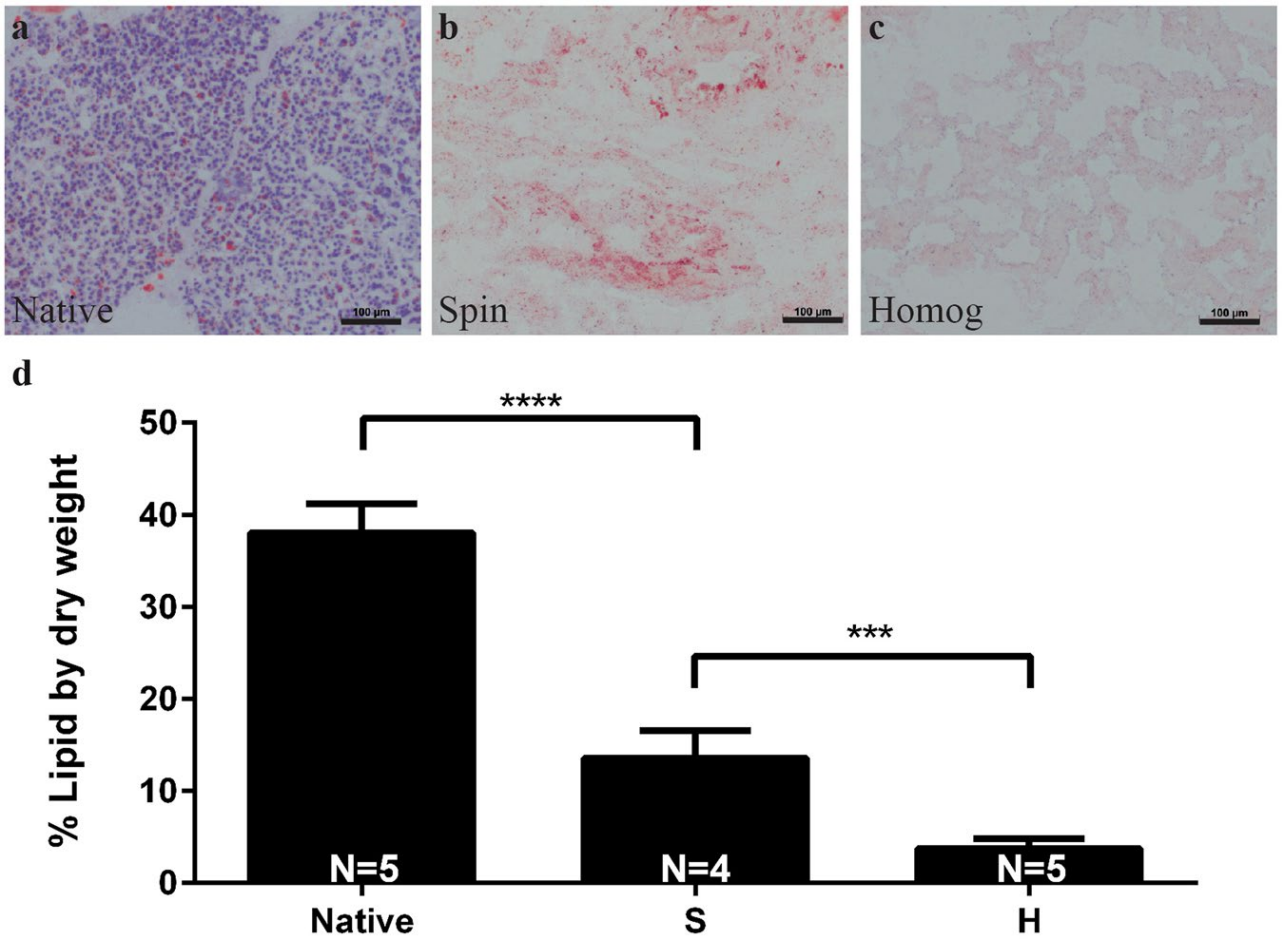
Delipidization was assessed by Oil Red O staining (ORO) on native, and decellularized tissues (**a–c**) and quantification of lipid content per mg dry weight utilizing the Folch method (**d**). (**a**) Native tissue contains a high amount of fat as shown by ORO staining, and tissue decellularized through spin (S) retains much of the fat content (**b**), while tissue decellularized with homogenization (H) has significantly reduced fat content (**c**). Scale bar represents 100 µm. N = 4–5 biological replicates per group.

Discarded, non-diabetic human pancreata were decellularized by either a spin-decell or a homogenization-decell protocol, as outlined in Fig. 1. The spin-decell protocol, in which 1 cm^3^ pieces of tissue are incubated in detergent, was compared to the homogenization-decell protocol, in which pancreas is first homogenized and centrifuged to remove extricated insoluble fat, and then incubated in detergent. The resulting human pancreatic ECM (hP-ECM) from both protocols was analysed for lipid removal (Fig. 2). We found that homogenization prior to deoxycholate treatment resulted in a much more complete lipid removal than was achieved by spin-decell. Lipid content was qualitatively assessed with Oil Red O staining (Fig. 2a–c) and quantified with a modified Folch protocol (Fig. 2b). We found on average that native undecelled donor pancreata contain 38 ± 3.6% lipids by dry weight. hP-ECM derived with the spin protocol contained 13.6 ± 3.0% lipids, whereas hP-ECM derived with the homogenization protocol contained 3.9 ± 1.1% (Mean ± SD) (Fig. 2d), significantly lower than without homogenization. An additional 5-hour lipase treatment (150 U/mL) was tested following the deoxycholate, but we found that lipase treatment did not significantly enhance delipidization and in some cases had a negative effect on hydrogel formation (data not shown), so was not pursued further.

Following decellularization, hP-ECM was lyophilized and pepsin digested in the preparation of a human pancreatic hydrogel (hP-HG) (Fig. 1). In addition to the generation of hydrogel, the decellularized hP-ECM material can be fabricated into 3D scaffolds with a variety of shapes for use in composite cell-matrix applications (Fig. S1).

### Turbidimetric gelation kinetics

**Figure 3:**
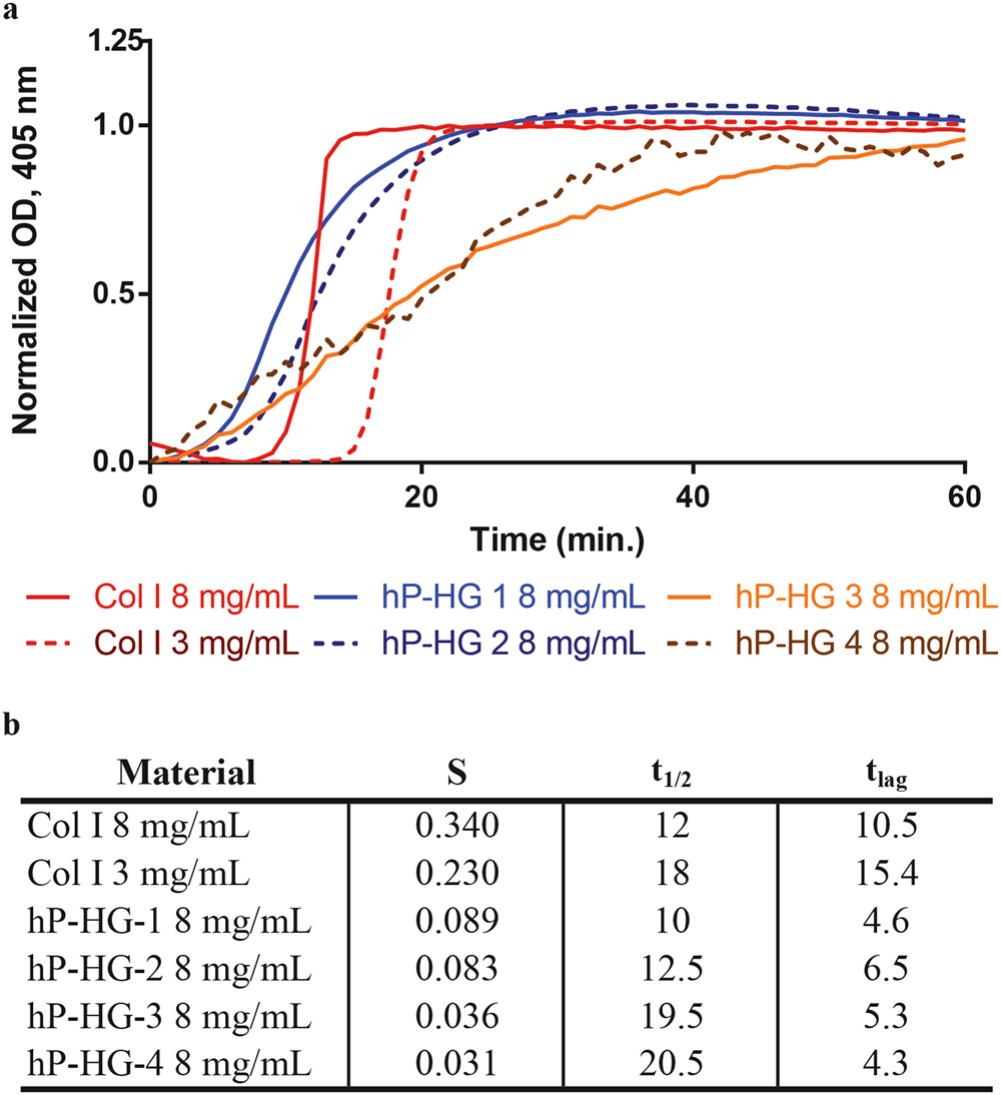
Representative turbidimetric gelation kinetics of collagen I and hP-HG. (**a**) Pre-gel solutions of collagen I, homogenization-decell (hP-HG 1&2) and spin-decell (hP-HG 3&4) were neutralized and added to the wells of a cold 96-well plate followed by incubation at 37 °C to induce gelation. (**b**) The t_1/2_ (time to 50% maximum absorbance), the speed of gelation (S) at t_1/2_ (indicates gelation rate) and the t_lag_ (time to upslope) calculated from the turbidimetric gelation curves are indicated for each material. N = 6 biological replicates for each protocol.

Gelation kinetics of hP-HG were characterised through turbidimetric analysis at a 405 nm wavelength and compared to that of purified collagen I hydrogel. This assay reads the absorbance of Collagen 1 fibrils throughout the process of hydrogel formation, and can be used for measurements of kinetics. hP-HG gels derived from homogenization-decell (Gels 1 and 2) were compared to gels derived from spin-decell (Gels 3 and 4). The normalized absorbance values of hP-HG alongside collagen I hydrogel are shown in Fig. 3a, along with the corresponding calculated parameters, shown in Fig. 3b. Homogenization-decelled hP-HG exhibited a sigmoidal curve in the gelation assay, similar to that of collagen I, but with a lag time of about 5–6 minutes, shorter than the 10–15 minute lag time of collagen. Despite the shorter lag time, the gelation speed (S) is slower than collagen gel, while the t_1/2_ itself was similar between collagen I and hP-HG. This indicates that hP-HG begins to gel more rapidly than collagen when heated to 37 °C, but takes the same overall time to reach complete gelation. In comparing hydrogels derived from the two described decellularization protocols, the spin-decell gels have a significantly slower gelation speed and a significantly longer time to t_1/2_. Further, these gels took longer to form and, in some instances, would not form gel at all.

Spin-decell hP-ECM generally contained visible fat in the interior of the decelled cubes, and following treatment with pepsin the digest remained cloudy and exhibited poor gelation characteristics (Fig. S2). We concluded from the lipid content and hydrogel turbidimetric data that the homogenization-decell protocol was more effective, demonstrating significant reductions in lipid content with superior hydrogel forming capability; therefore, all subsequent analyses focused on hP-ECM derived with the homogenization protocol, as well as hydrogel produced from this homogenized ECM.

### Characterisation of hP-ECM and hP-HG

**Figure 4:**
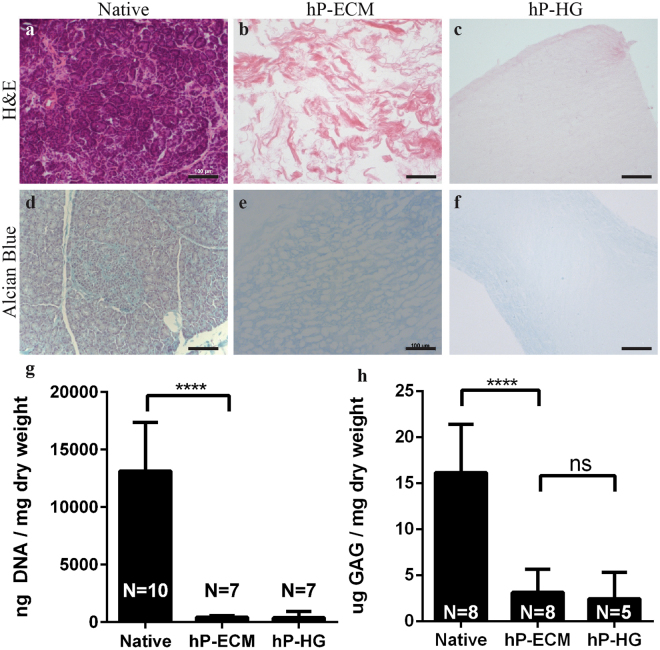
Characterisation of pancreas decellularization. Representative images of human pancreas decellularization efficiency assessed qualitatively by H&E before and after decellularization (**a–c**) and quantitatively by PicoGreen Assay (**g**) in ng of DNA/mg dry tissue weight. Histologic evaluation by Alcian Blue (sGAG, blue) staining (**d–f**) and quantitatively (**h**) with biological analysis of ECM sGAG content normalized to ECM dry weight. Scale bar represents 100 µm. ****p < 0.0001, ***p < 0.009.

The ECM and hydrogel obtained by the homogenization-decell protocol were analysed for retention of DNA, sGAG and common ECM proteins. hP-ECM and hP-HG are devoid of nuclei as shown by H&E staining (Fig. 4b and c) compared to native pancreas (Fig. 4a). The DNA content of the decellularized pancreas was reduced to only 3.6% of the DNA content of native tissue, indicating that homogenization and deoxycholate treatment was successful at removing DNA (Fig. 4g). The DNA content of hP-HG contained only 3.1% of the native tissue amount.

GAG content for each sample was analysed using qualitative and quantitative methods to determine GAG retention following decellularization and hydrogel formation. The histochemical stain Alcian blue was used to visualize sGAG, molecules that participate in biochemical signalling, play structural roles within the ECM, and support hydration of ECM and hydrogels. Histological examination of native pancreas shows positive sGAG staining throughout the parenchyma and more concentrated staining within islets. sGAG content appears to be retained in both hP-ECM and hP-HG as demonstrated by the presence of blue stain in the matrix (Fig. 4d–f). However, quantitative sGAG assessment demonstrated a significant decrease in content following the decellularization procedure. hP-ECM was found to retain 19.6% of the native sGAG content, while hP-HG was found to only retain 15.2% (Fig. 4h).

**Figure 5:**
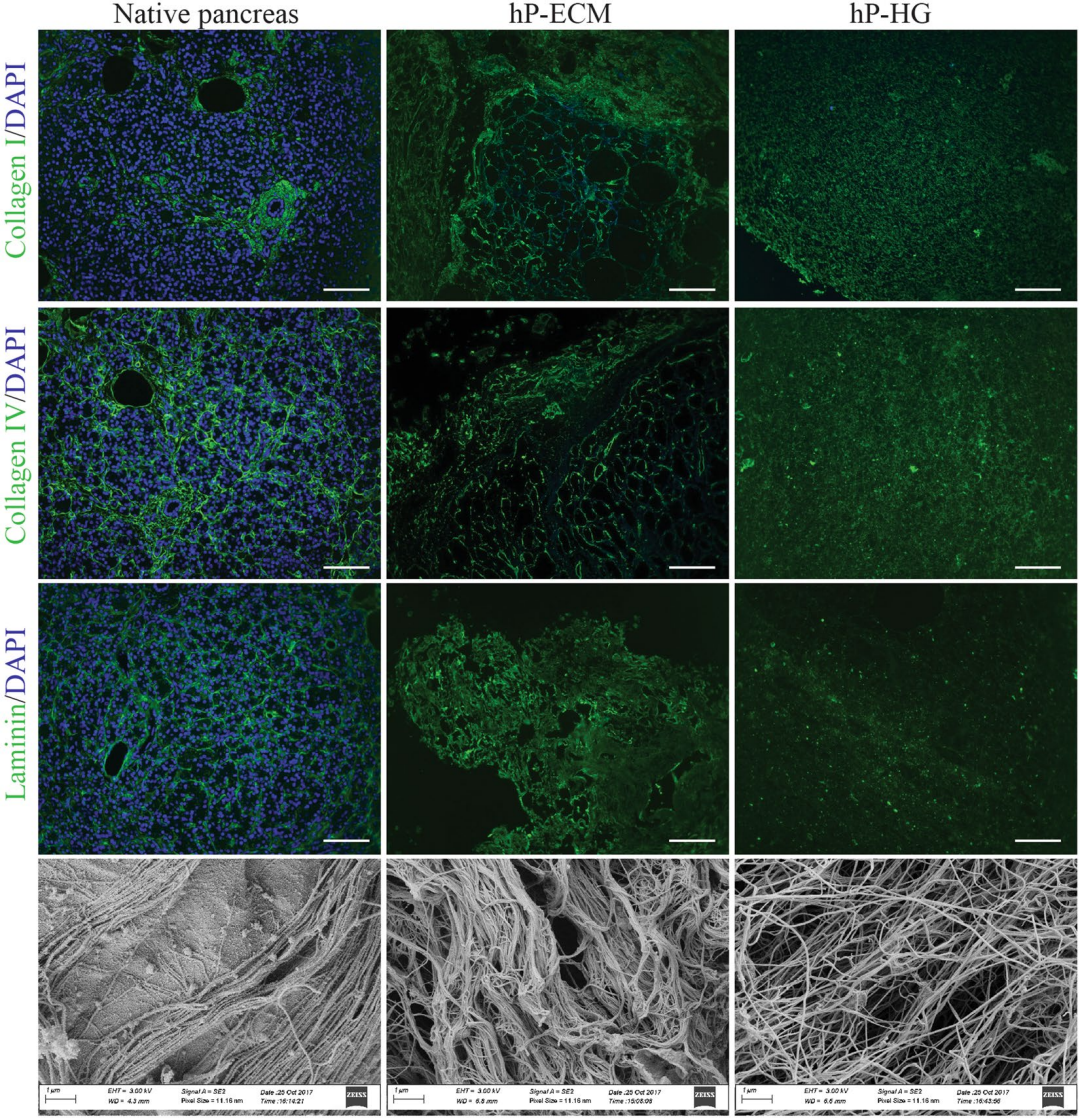
Histological and morphological analysis on native tissue and on decellularized pancreatic hydrogels. ECM protein retention was assessed by IHC in native pancreas (left column), decellularized scaffold (middle column) and hydrogel (right column) for: collagen I (top 3 panels); Collagen IV (upper middle panels) and laminin (lower middle). Scanning electron microscopy (bottom row) of native (left) and hP-ECM (middle) and hP-HG (right). SEM images of scaffold and gel reveal a porous 3D structure composed of intermeshed fibres. Images were obtained at 10,000x magnification. Scale bar represents 100 µm.

To further characterise the decellularized pancreatic ECM and the resulting hydrogel, immunofluorescent (IF) detection of ECM proteins was performed. We found that collagen I, collagen IV, and laminins are retained in decellularized materials, and the ECM microarchitecture appears to be preserved within the homogenized hP-ECM (Fig. 5).

**Figure 6:**
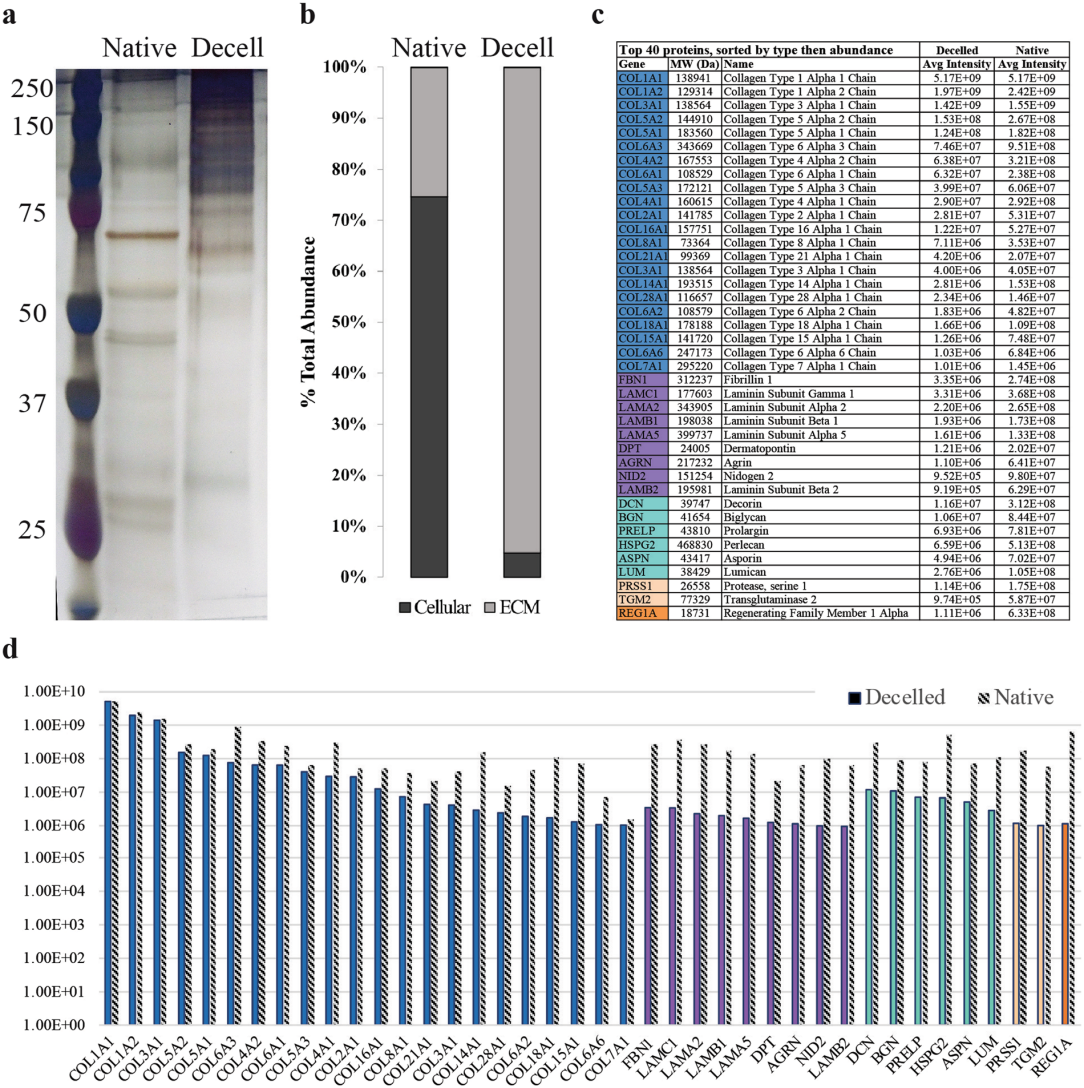
Protein content of native and decellularized pancreata. (**a**) A representative silver stained SDS-PAGE comparing total protein (10 µg) content of native and decellularized pancreas demonstrates enrichment of large molecular weight proteins (80–250kD), and (**b**) MS-based analysis of the percent total abundance represented by ECM-associated or cellular proteins in the native and decellularized pancreas. (**c**) A list of the top 40 ECM-associated proteins identified in the decellularized material, sorted first by category (blue = collagens, purple = ECM glycoproteins, teal = proteoglycans, beige = ECM regulators, orange = ECM-affiliated proteins) and second by normalized abundance in the decelled sample, (**d**) graphed alongside the normalized abundance in the native tissue (grey striped bars). N = 3 biological replicates per group analysed for SDS-PAGE/silver stain analysis.

To assess matrix ultrastructural morphology, native pancreatic tissue, decellularized hP-ECM and hP-HG were subjected to scanning electron microscopy (SEM). SEM images display retention of 3D ECM architectural elements as well as preservation of fibrillar structures of the native ECM. These organised fibrillar bundles appear to be preserved through the decellularization and delipidization process which are likely to be the fibrous collagen proteins identified by MS as the top 22 ECM-associated proteins (see Fig. 6). The decellularized ECM material contains visibly apparent negative space. This space may be the “footprint” previously occupied with native cells, and provides a physical space for exogenous cells to take up residence and receive direction from the biological components remaining in the ECM scaffold. hP-HG also displays reformed ECM fibrils by SEM. Additional high-magnification images are included in Supplemental Fig. S3.

Having demonstrated the retention of several major ECM proteins after decellularization by IF, we sought to visualize the change in overall protein composition following this procedure. Protein samples from native pancreas and hP-ECM were run on an SDS-PAGE gel and probed with a silver stain to visualize total protein content. With equal total protein loaded for each sample, the presence of higher molecular weight proteins in the decellularized sample compared to native tissue is apparent, revealing that the large molecular weight ECM proteins^34^ have been enriched in the acellular material (Fig. 6a). These bands most likely represent the two main classes of macromolecules, the fibrous proteins such as collagens and the glycoproteins such as laminins, which due to being enriched following dellularization are now detectable, as many other low-abundance proteins are not detectable with this method. To more thoroughly evaluate the retention of the ECM proteins after decellularization, isobaric tagging for multiplexed quantitation using custom-developed dimethylated leucine (DiLeu) labels was employed to enable high-throughput quantitation of relative abundances of all proteins by high-resolution mass spectrometry. All identified proteins were categorized as either ‘cellular’ or ‘ECM’ based on the MatrixDB database^35^. Native pancreatic tissue was found to be composed of 74.5% cellular proteins and 25.4% ECM proteins, while decellularized ECM contained only 4.8% cellular proteins and 95.2% ECM proteins (Fig. 6b). The top 40 most abundant ECM-related proteins in the decellularized and delipidized hP-ECM are listed in Fig. 6c, and graphically represented in Fig. 6d. The average signal intensity of each peak is listed in the table, and the intensity of the corresponding protein in the native (i.e. non-decelled) sample is included next to each bar on the graph in grey (Fig. 6d). A complete list of all 120 ECM-related proteins identified in the samples is included in Supplemental Fig. S4.

### *In vitro* cytocompatibility

hP-HG has potential for use as scaffolding or substrate material for *in vitro* cell culture, where the pancreatic ECM proteins may provide a microenvironmental niche to improve survival of islets or stem cell-derived beta-like cells. We aimed to verify the cytocompatibility of hP-ECM with a variety of relevant cell types including an insulinoma cell line, stem cell-derived beta-like cells, and endothelial cells, which could ultimately aid in graft vascularization.

First, we investigated the ability of the hP-HG to support cell adhesion and survival in 2D cell culture using INS-1 832/13 cells (stably transfected rat insulinoma cells engineered to express human insulin)^36^ and human umbilical vein endothelial cells (HUVECs). Each cell line was plated on untreated, hP-HG-coated, and collagen I-coated plates, and interrogated with an MTS metabolic activity assay and live/dead staining on days 1, 2, 3 and 4 after plating. Growth curves were generated for each cell line grown on each substrate. Regardless of substrate, growth curves were indistinguishable from one another for both INS-1 832/13 cells and HUVECs (Fig. S5a,b). In addition, the INS 832/13 cells plated as described above, were subjected to glucose stimulated insulin secretion assays (GSIS) to determine if the glucose responsiveness was maintained in all experimental groups. The stimulation index, a marker for insulin secretion functionality, calculated for all platforms was 2.48 ± 0.78, with no group being statistically different from another. This indicates that the cells were functional on all platforms tested. Furthermore, the live/dead staining revealed an insignificant difference in the ratio of live:dead cells for all cells and conditions after 4 days in culture. Importantly, cells grown on hP-HG maintained their cell fate, as evaluated through IF staining for human insulin and rat NKX6.1 expression in the INS-1 832/13 cells and for vWF and E-Cadherin expression in HUVECs (Fig. S5c–f). Additionally, HUVECs maintained expected expression of CD31 (data not shown). Together, these results demonstrate that cells grow equally as well on hydrogel-coated surfaces as on untreated plastic or Col I in short-term culture, and that hP-HG does not induce significant cell death or inhibit cell growth.

**Figure 7:**
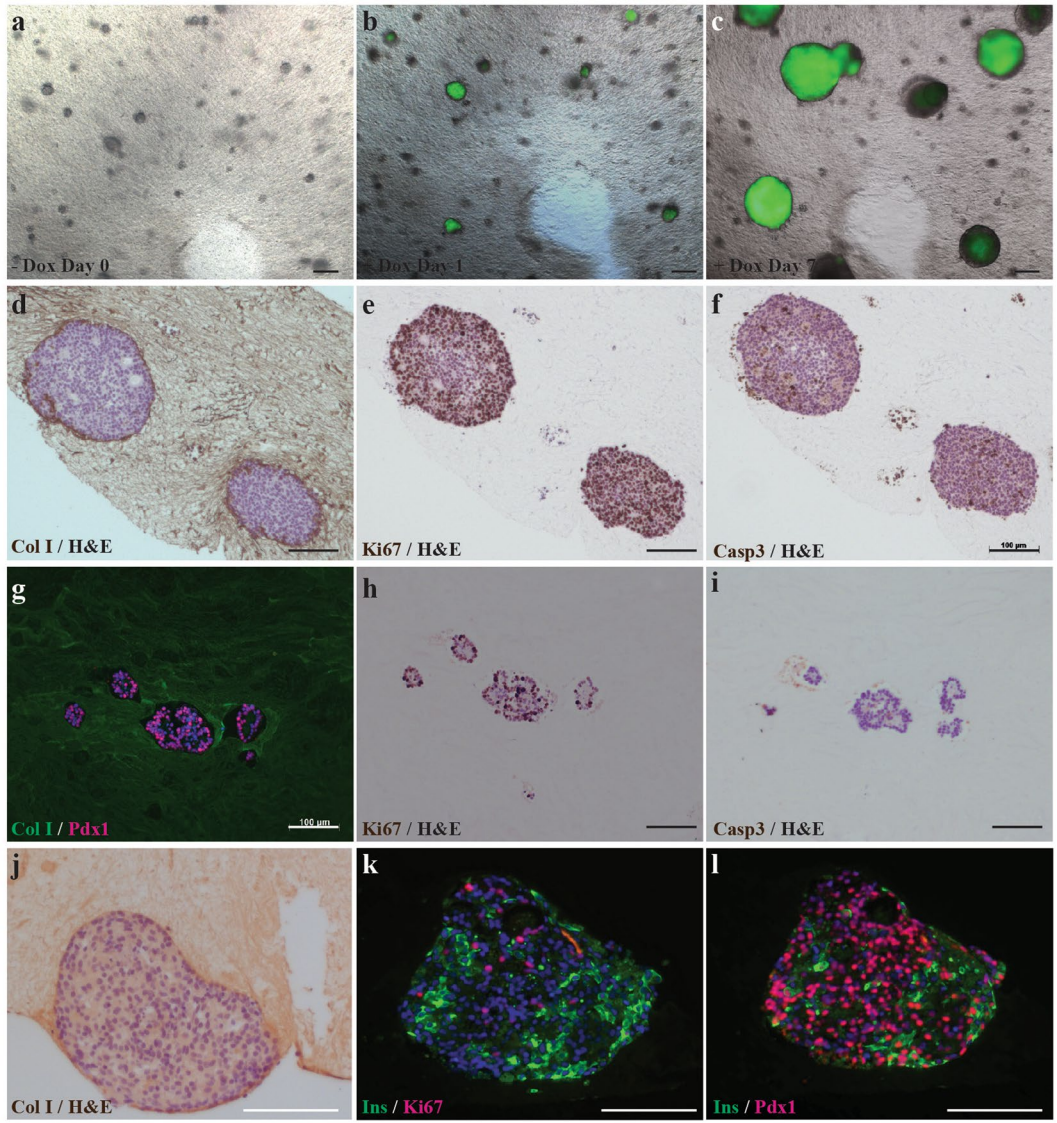
*In vitro* cytocompatibility assessment. Doxycycline inducible GFP H9 hESC clusters were embedded in hP-HG. (**a–c**) Cells were serially imaged using epifluorescence in live cultures on days 0, 1, and 7 after Dox treatment (1.5 µg/ml in E8 media). Images show progressive expansion of green fluorescing colonies in 3D indicating survival and growth of hESCs. (**d–f**) At day 7 of culture, undifferentiated expanded Dox-GFP H9 colonies were fixed, paraffin embedded, and stained with H&E and counterstained for either Col I, Ki67 or Caspase 3 (Casp3). The images show hESC colonies embedded in hP-HG matrix highly express Ki67 proliferation marker and few Casp3 expressing cells. (**g–i**) hPSC- derived pancreatic progenitors embedded in hP-HG and cultured for 9 days and fixed, embedded in paraffin, sectioned and stained for Col I (green) and PDX1 (red nuclei). Pancreatic progenitors embedded in hP-HG also express Ki67 indicating expected proliferation, but minimal Caspase 3 positive staining. (**j–l**) hPSC-derived ILCs were embedded in hP-HG for 4 days, fixed, embedded, and stained for Col I, Ins (green), Ki67 (red) or PDX1 (red nuclei). Images show healthy cell clusters containing non-proliferative Ins+ cells in the clusters and numerous PDX1^+^ Ins^−^ progenitor cells and Ins^+^ PDX1^+^ beta-like cells. Scale bar represents 200 µm in (**a–c)** and (**j–l)**, and 100 µm in (**d–i)**. Representative images shown, N = 3 biological replicates for all groups.

To test 3D culture compatibility with hESCs, doxycycline-inducible (Dox) GFP-expressing H9 stem cells were embedded as small colonies in hP-HG and maintained in E8 growth medium for 7 days in the presence of Dox, which was initiated 24 hours after plating. After 24 hours of Dox exposure, the cells were imaged and GFP was visible. After 7 days of Dox treatment, GFP expression and expansion of colonies in 3 dimensions was apparent as demonstrated with live imaging (Fig. 7a–c). At the experimental end point the hP-HG embedded cell colonies were fixed and analysed for Ki67 and Caspase 3 marker expression to assess proliferation and apoptosis, respectively (Fig. 7d–f). These cells were found to be on average 94.0% positive for Ki67 and 3.6% positive for Caspase-3, indicating that undifferentiated H9 cells can expand and grow while embedded in hP-HG. This demonstrates that hP-HG is cytocompatible with the human stem cell line H9, non-toxic and permeable to small molecules such as Dox. Additionally, the H1 hESC line and human iPSC lines derived from CD34^+^ cells have been combined successfully with hP-HG for short-term undifferentiated expansion (data not shown).

To test for compatibility of hP-HG with hESC-derived cells, pancreatic progenitor cells (differentiated for 11 days), were embedded in hP-HG, cultured in appropriate stage-specific medium, and stained for the stage-specific marker PDX1, as well as Ki67 and Caspase-3 to assess proliferation and apoptosis, respectively, in the embedded cells (Fig. 7g–i). Further, we investigated cytocompatibility with hESC-derived pancreatic endocrine cells or islet-like clusters (ILCs) (following a stepwise differentiation protocol for 28 days), which were embedded in hP-HG, cultured in end stage differentiation medium for 4 days, and analysed for insulin, PDX1, Ki67 and Caspase 3 (Fig. 7j–l). Quantification of the differentiated cells co-cultured with hydrogel (H), compared with cells that remained in suspension (S), showed insignificant differences in the percentage of cells which were Ki67^+^ (H: 36.3%, S: 32.5%), Caspase-3^+^ (H: 2.7%, S: 2.4%), and PDX1^+^ (H: 77.5%, S: 74.7%). These studies support the notion that hP-HG supports the growth and maintenance of cell fate of hESC-derived pancreatic progenitors and ILCs and provide a suitable substrate for *in vitro* culture.

### *In vivo* immunogenicity

**Figure 8:**
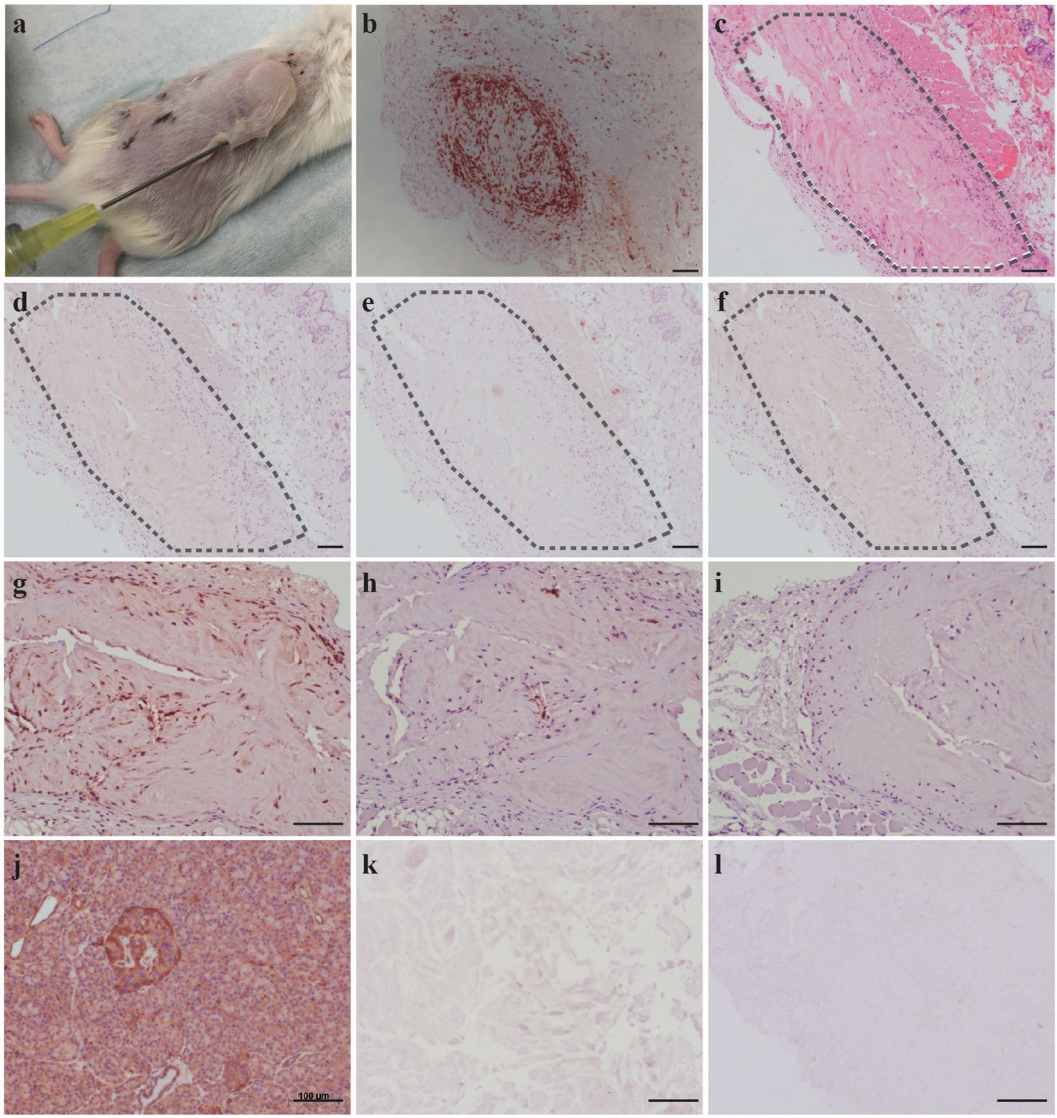
Minimal immune cell responses to decellularized human pancreatic matrix in humanized mice. (**a**) 500 µl of hP-HG was injected subcutaneously on one flank and allogeneic human foetal pancreas (HFP) tissue was implanted into the opposite flank of the same humanized mouse. The grafts were collected after 4 weeks and analysed for histology and immune cell infiltration. (**b**) HFP showed intense hCD3 immune infiltration suggesting an acute cellular rejection response. (**b–l**) The hP-HG hydrogel graft was analysed by: (**c**) H&E, and by staining for cellular phenotypes including: (**d**) CD3^+^ T cells, (**e**) CD8^+^ T cells, (**f**) FoxP3^+^ regulatory cells. The hP-HG graft was also stained for: (**g**) mouse specific CD68^+^ macrophages, (**h**) human specific CD68^+^ macrophages, and (**i**) human specific CD20^+^ B cells. The graft appears to be non-immunogenic as the graft is largely negative for CD3^+^ and CD8^+^ T cells and CD20^+^ B cells. The majority of the infiltrating cells are mouse CD68^+^ cells with a few scattered human CD68^+^ cells present (**h**). (**j–l**) IHC staining of native (**j**) and decellularized pancreas tissues hP-ECM (**k**) and hP-HG (**l**) for pan human HLA-ABC show that the decellularized ECM and hydrogel are negative for HLA Class I antigen expression. Scale bar represents 100 µm.

In order to utilize this material for biomedical applications such as allograft tissue engineering, it is important to assess whether hP-HG induces an immune response upon transplantation. The major goal of decellularization protocols is to remove the cellular and antigenic material efficiently while maintaining the important ECM molecules, which are themselves inherently hypoimmunogenic^37^. To test the immune response to hP-HG, we employed immunodeficient NSG mice endowed with a humanized immune system using human foetal thymus and CD34^+^ hematopoietic stem cells. After 12 weeks, allowing for multi-lineage haematopoiesis to occur, the mice exhibited greater than 25% engraftment of human CD45^+^ cells. The humanized immune system is derived from foetal tissue distinct from the adult donor pancreas with which the hP-HG was derived. Therefore, the humanized immune system should recognise and attack hP-HG if it represents immunogenic material. To test the immunogenicity, a bolus of neutralized hP-HG pre-gel solution was injected subcutaneously into the dorsum of humanized mice, allowing it to gel *in vivo*. As a positive control for graft rejection, human foetal pancreas (HFP) tissue from another individual, allogeneic to the foetal tissue used to produce the humanized mouse, was also implanted in the same animal subcutaneously. Grafts were removed after 4 weeks to assess immune response. H&E staining shows strong infiltration of inflammatory cells into the foetal pancreas tissue while the hP-HG shows very little immune cell infiltration. Further immunological evaluations for human CD3 and CD8 were performed and revealed the HFP tissue to be highly infiltrated with cytotoxic T lymphocytes, while hP-HG graft contained little to no infiltration of similar human immune cells (Fig. 8b,d and e).

In the native pancreas, IHC staining indicates expression of HLA class I proteins, while staining of decellularized hP-ECM and hP-HG reveals the absence of HLA class I antigens (Fig. 8j–l). The same trend was seen following interrogation of HLA Class II antigens (HLA-DR) (data not shown). Thus, the *in vivo* assessment of hP-HG suggests it is hypoimmunogenic in this humanized mouse model consistent with the notion that the cellular and antigenic components of the tissue have been largely removed.

## Discussion

Decellularized tissues from a variety of sources have been utilised in human clinical applications, primarily for soft tissue reconstruction^38^. Natural organ ECM represents an important substrate formed from a complex of highly ordered fibrous proteins which provides many biological properties known to support cell viability and growth. Whole organ decellularized pancreatic ECM has been derived from mice, rats, pigs and humans^39–45^ as a potential alternative to traditional organ transplantation where relevant cell types are reseeded into the decellularized matrix with the hope of reconstituting a neo-organ with functional properties. However, recellularizing and rebuilding the vasculature of intact decellularized organ scaffolds can be challenging^39,41,43,45–47^. An alternative approach, which is particularly relevant for an islet endocrine graft, would be to combine cells with ECM-derived hydrogel. Such a strategy is advantageous for tissue engineering applications because of its ease of use.

ECM hydrogels have been the focus of research for a number of years and have been developed from a variety of tissue sources including adipose^12,22,48^, urinary bladder^49^, liver^50^, nervous system^51^, dermis^52,53^, skeletal muscle^54^ and cardiac tissues^55–57^. However, this has not yet been achieved with human pancreas. The human pancreas is an organ that has a relatively high lipid content, which is inhibitory to the production of a hydrogel, as similarly described by Young *et al*. in processing adipose tissue to hydrogel^12^. We found that many human donor pancreata contain a substantially high lipid content, which is due to adipose tissue sequestered throughout the parenchyma of the organ. Notably, we have received some human pancreata which contained up to 70% lipid content by dry weight. This excess lipid prevented successful solubilisation and gelation of the hP-ECM. In applying published animal pancreas decellularization methods to human pancreas, we were not successful in making hydrogel, indicating that further optimization of the protocol was required to remove more lipids. Therefore, to eliminate these lipids from the matrix more effectively, we included a unique homogenization step in the decellularization protocol. This method significantly aided in fat removal from the homogenized matrix, thereby allowing for the efficient generation of hP-ECM scaffolding and hydrogel, with preserved ECM proteins in an arrangement similar in architecture to native pancreas. Following effective delipidization, we demonstrated that a hydrogel can be formed which has similar gelation kinetics to collagen I, as demonstrated by a turbidimetric gelation assay. We also showed that the hP-HG is cytocompatible with a number of cell types in *in vitro* culture applications and does not elicit an immune response *in vivo* in humanized mice, supporting its potential in pancreatic endocrine tissue engineering applications.

ECM is comprised of many protein and polysaccharide macromolecules including collagens, laminins, proteoglycans and more. Retention of native ECM proteins is essential in order to reproduce the pancreatic ECM niche and facilitate cell attachment and ECM signalling^58^. We showed through IF staining that collagen I, collagen IV and laminins were retained in the decellularized matrix and that the microstructural organisation of these proteins was maintained throughout the decell process, even following homogenization (Fig. 4). Collagen I and collagen IV were retained in the digested hydrogel, while laminin appeared as fragments within the gel. This may be due to the fact that laminins are susceptible to pepsin digestion, and potentially reduced due to the cleaving antibody binding sites. However, results from several studies suggest that following pepsin digestion, laminin fragments still retain growth factor and integrin binding capabilities and therefore may maintain function^59–61^.

It is well established that sulphated GAGs are an important component of the ECM, through their involvement in growth factor sequestration and presentation^62–64^. Development of a decellularization/delipidization protocol that maintains these macromolecules is ideal in the production of a material that presents an *in vivo*-like niche. ECM components were expected to be retained in the matrix and hydrogel to varying degrees of efficiency; we found that GAGs were partially retained in the decellularized matrix and the digested hydrogel. It has been established that pancreatic beta cells contain a significant level of intracellular sGAG,^65^ which is consistent with our findings that *in situ* islets are enriched for cellular sGAG (Fig. 4). This may be one contributing factor to the large reduction in sGAG content, as these molecules would be removed along with the cellular material during decellularization. Furthermore, while pepsin digestion in the process of hydrogel formation may result in an additional, unavoidable, reduction in GAG content, it may be possible to infuse or crosslink the gel with additional GAGs or GAG mimetics to enhance cell signalling and attachment^66^. Further investigation toward delineating the specific roles individual GAGs play within the islet ECM niche will be necessary to fully understand and elucidate the importance of GAGs within this material.

DiLeu isobaric tandem mass tags coupled with high resolution mass spectrometry was employed for high throughput proteomic analysis of the human pancreatic matrisome. This approach has a broad dynamic range, offering accurate and highly sensitive quantitative analysis of protein content. Pooling of labelled native and decelled samples enriches the mixture for ECM proteins and permits detection of low-abundance proteins that may not otherwise been distinguished. 120 ECM and ECM-associated proteins were identified in the hP-ECM, which is four times the number of ECM proteins described in the mouse decelled pancreas^40^ and tenfold greater than what has been identified in the porcine pancreas^67^. Moreover, this is the first reported list of ECM proteins quantitatively measured in the human pancreas by mass spectrometry, enabling assessment of the relative abundance of the various ECM components.

Generally, collagens were observed to have the best retention following the decellularization treatment, with the intensity most closely matching that found in the native tissue. While proteoglycans and glycoproteins have reduced total abundance in the decelled material as compared to the native tissue, many of them increase in rank abundance, meaning that relative to other ECM proteins, they are more enriched following decellularization. We identified proteoglycans of several different families, including those which localize to the pericellular/basement membrane or extracellular space and also including proteins belonging to the heparan sulphate, keratan sulphate, chondroitin sulphate and dermatan sulphate-binding families^68^. The identification of these pancreas-specific ECM proteins and proteoglycans will allow for future testing of their presence and properties within hP-HG scaffold constructs, and their contribution to pancreatic cell fate determination.

To assess the biocompatibility of the hP-HG *in vivo*, immunogenicity studies were conducted by utilizing humanized mice. When injected subcutaneously, hP-HG did not elicit an immune response, while allogeneic HFP tissue transplanted into the same animals was acutely rejected, as expected. Immune reactions occur with incompatibility between ABO and HLA systems, mainly triggered by the recipient in response to the donor^69^. The IHC performed for HLA class I and II (HLA-ABC/-DR) in native pancreas, hP-ECM and hP-HG clearly demonstrates the removal of these antigens from the decellularized materials, further corroborating the absence of an immune response to the acellular material in the humanized mice. The results suggest this hypoimmunogenic pancreatic hydrogel, when transplanted with cells or islets, may not illicit immune rejection in an *in vivo* environment.

hP-HG is a versatile product that can be utilised in a variety of tissue culture and transplantation applications. The pancreatic hydrogel proved to be cytocompatible with a variety of cell types indicating that it permits normal growth of cells and appears to be non-toxic. We observed that INS-1 832/13 cells and HUVECs both grew equally well in 2D culture on untreated, collagen I-coated, and hP-HG-coated tissue culture plates. These results translated nicely into the use of hP-HG in 3D culture where we challenged undifferentiated H9 cells, as well as stem cells differentiated toward a beta cell fate, to survive and grow in these matrix modules. When undifferentiated human stem cells were embedded in hP-HG, they expanded in a 3D spherical pattern. These cells could be induced to express GFP with media containing doxycycline, indicating that the hydrogel is permeable to small molecules and other media components. Likewise, cells differentiated toward a pancreatic endocrine fate survived and retained their identity when embedded in hydrogel, as demonstrated through continued expression of known endocrine markers such as PDX1. Future work will consist of investigating the effect hP-HG may have on differentiating stem cell-derived islet-like cells cultured within the gel, compared to cells differentiated without exposure to hP-HG. We aim to test whether hP-HG has the capacity to improve the differentiation, maturation and function of these cells *in vitro*.

The results from this study illustrate that discarded human pancreata can be successfully decellularized, delipidized and processed for development of 3D scaffold casts and hydrogels which maintain their macromolecules, are not toxic to the growth and differentiation of several types of cells and therefore may have value in regenerative medicine applications. *In vivo*, cells are in contact with the unique ECM of the tissue they reside within, providing tissue-specific signals which aid in cell fate determination. Therefore, including these tissue-specific cues in an *in vitro* cell culture system will likely enhance the culture environment in ways that synthetic or purified individual ECM components cannot achieve alone. Studies are ongoing to determine the specific components and properties of the pancreatic ECM in order to establish a useful scaffold for the growth and maintenance of stem cell-derived beta cells and cadaveric islets for transplantation.

## Materials and Methods

All experiments were performed using protocols approved by the Animal Care and Use Committee of the University of Wisconsin School of Medicine and Public Health and the Health Sciences Institutional Review Board, and complied with federal and state law.

### Pancreata Procurement

Human cadaver pancreata were procured for either research or transplantation. If recovered for transplantation but ultimately deemed unusable, they were earmarked for research. No organs were procured from prisoners. Human pancreata (n = 11, age 13–58 years) were obtained and used in this study through the University of Wisconsin Organ and Tissue Donation with consent obtained for research from next of kin and authorization by the University of Wisconsin-Madison Health Sciences Institutional Review Board and was performed in accordance with federal and state law. A list of donors used in this study and their demographic data are shown in Supplemental Table 1. (More details can be found in Supplementary Methods).

### Spin-Decellularization (Spin-decell)

Human pancreata were trimmed of surrounding fat, and the remaining parenchyma was sectioned into approximately 1 cm^3^ pieces, flash frozen and stored at −80 °C. For decellularization, tissue was thawed at 37 °C until soft, rinsed with 1X PBS for 30 minutes, and placed in 2.5 mM sodium deoxycholate/PBS, shaking at RT for 24 hours. The tissue was then rinsed with water and the 24-hour deoxycholate shake was repeated. After a total of 48 hours of agitation in deoxycholate, the tissue was rinsed with water and washed in 1X PBS supplemented with 1X Pen/Strep for 72 hours, with water rinses every 24 hours, and fresh PBS+ Pen/Strep replaced each day. The resulting decellularized pancreatic ECM was lyophilized and stored at −80 °C for future use.

### Homogenized-Decellularization (Homogenization-decell)

Human pancreata were processed as above until thawed. The tissue was rinsed with 1X PBS for 30 minutes, washed with water and homogenized in water until broken up. The homogenate was centrifuged (4300 rpm, 5 min), floating fat was removed from the surface, and the cloudy supernatant was discarded. The pellet was resuspended in water and centrifuged again (4300 rpm, 5 min). The pellet was then resuspended into 2.5 mM sodium deoxycholate/PBS and incubated for 3 hours (RT, shaker). After this time, the homogenate was strained over a sieve (Sigma, S1145); all collected material was placed back into 2.5 mM sodium deoxycholate/PBS and incubated for an additional 15 hours (RT, shaker). The ECM was strained again, rinsed with water and washed in 1X PBS supplemented with Pen/Strep for 72 hours, with water rinses every 24 hours, and fresh PBS+ Pen/Strep replaced each day (RT, shaker). The resulting decellularized pancreatic ECM was lyophilized and stored at −80 °C for future use.

### Hydrogel Formation

Lyophilized ECM was digested with an HCl/pepsin solution and then neutralized, as previously described^49^. Controls were made by neutralizing rat-tail collagen type I (Corning, 354249). (More details can be found in Supplementary Methods).

### Hydrogel coated plates

Acidic ECM digest was diluted with cold 1X PBS to a concentration of 0.08 mg/ml (a 1:120 dilution) and filtered through a 40 µm filter to remove undigested ECM pieces. 300 µl of diluted digest (pre-gel solution) was added to each well of a chilled 24-well culture plate (Corning, 3527). Plates were incubated at 37 °C for one hour and rinsed with PBS before use.

### Matrix characterisation

#### DNA Content

Quantification of DNA was assessed using the Quant-iT™ PicoGreen® dsDNA Assay (Life Technologies, P7589), following manufacturer’s protocol. Weighed and lyophilized ECM materials were digested with papain for 3–18 hours at 65 °C prior to the assay.

#### Sulphated Glycosaminoglycan (sGAG) Content

The sulphated glycosaminoglycan (GAG) content of native and decellularized pancreata was quantified using the Blyscan GAG Assay Kit (Biocolor, UK), following manufacturer’s protocol. Weighed and lyophilized ECM materials were digested with papain for 3–18 hours at 65 °C prior to the assay. The absorbance at 595 nm was measured using a microplate reader (FlexStation 3, Molecular Devices) and compared to standards. Absorbance values were normalized to sample dry weight.

#### SDS-PAGE analysis

Protein lysates were prepared from native pancreas tissue, decellularized/lyophilized hP-ECM and hP-HG digest solution. Samples were solubilized in 1X RIPA buffer + Halt protease inhibitor cocktail (ThermoFisher, 78430) and total protein content was measured with a DC Protein Assay (BioRad, 5000112). To compare protein distribution, 10 µg of protein from each sample was resolved on a 10% SDS-PAGE gel (Biorad, 3450111) and silver-stained for visualisation.

#### Lipid Content

Samples were embedded in OCT and cut into 5 µm sections. Sections were fixed with 10% neutral buffered formalin for 5 minutes, rinsed with water and then 70% ethanol. Slides were incubated for 10 minutes in Oil Red O (ORO) stain, washed with 70% ethanol and counterstained with haematoxylin. Images were generated with a Zeiss Axiovert 200 M microscope for a qualitative evaluation of lipid retention. Quantitative lipid content was assessed using a modified Folch lipid extraction (See Supplementary Methods). The resulting lipid material was weighed to determine lipid content of the original sample.

### Histology and Immunofluorescence (IF) Microscopy

#### Basic Histology

Native and decellularized samples were fixed in 4% paraformaldehyde (PFA), paraffin embedded, and sectioned (5 µm) for histological examination. Sections were stained with haematoxylin and eosin (H&E). Additional sections were stained with Alcian Blue (AB), following standard protocols.

#### Immunohistochemistry

Slides were deparaffinized using xylene and rehydrated. Antigen retrieval was performed by treatment with 10 mM Citrate Buffer, pH6.0 for 2 hours in an 80 °C water bath. Slides were blocked with 10% BSA/PBS for 1 hour at RT, incubated with primary antibodies overnight at 4 °C, washed, incubated with secondary antibody incubation for 1 hour at RT and cover slipped.

The antibodies and dilutions are listed in Supplementary Table 2. Immunofluorescent secondary antibodies were Alexa Fluor 488 and 568 of anti-goat, anti-mouse, anti-rabbit. Nuclei were counterstained with 40–6-diamidino-2-phenylindole (DAPI). Images were generated with a Zeiss Axiovert 200 M microscope. Images were analysed and counted using Image J software. Total Ki67-positive cells were divided by total nuclei to calculate percentage of proliferative cells. Total Casp3-positive cells were divided by total nuclei to calculate the percentage of apoptotic cells. Total Pdx1-positive cells were divided by total DAPI-positive nuclei to calculate the percentage of Pdx1^+^ cells. At least 3000 total nuclei were counted across images in each group.

#### Turbidimetric-Kinetic Gelation Assay

Turbidimetric gelation kinetics were determined as previously described^49^. 100 µL of cold, neutralized hydrogel (pre-gel) was loaded per well into a flat-bottom 96-well plate, placed in a pre-heated (37 °C) FlexStation 3 (Molecular Devices) plate reader and the OD at 405 nm was recorded in 30-second intervals over the course of 80 minutes. Data was collected using SoftMaxPro software. Two biological replicates were performed in triplicate and averaged. Data was normalized using Equation 1, where A is the absorbance at a given time, A_0_ is the lowest absorbance (time zero) and A_max_ is the maximum absorbance.

1 $$Normalized\,Absorbance\,(NA)=\frac{{\rm{A}}-{{\rm{A}}}_{0}}{{A}_{max}-{A}_{0}}$$

The time needed to reach 50% of the maximum turbidity absorbance value was defined as t_1/2_; the lag phase (t_lag_) was calculated by extrapolating the x-intercept of the linear portion of the curve. The speed of gelation (S) was determined by calculating the slope of the curve at t_1/2_ as described in^54^.

#### Scanning Electron Microscopy

Samples were fixed overnight in 4% PFA, washed and stored in 1X PBS. Samples were progressively dehydrated through a series of ethanol washes starting at a 30% solution and ending with 100% ethanol. Samples were dried on a critical point drier and coated with a 40:60 mixture of gold:palladium. Images were taken with a LEO 1530 scanning electron microscope at 1000X, 2500X, 10000X and 40000X.

### *In Vitro* Cytocompatibility

#### Live/Dead Imaging

HUVECs (1.6 × 10^4^ cells/cm^2^) (Sigma, 200P-05N) and INS-1 832/13 cells (2.6 × 10^4^ cells/cm^2^)^36^ were plated on 24-well plates with either no coating, collagen I coating (Corning, 354249) or hP-HG coating, and cultured for 1–3 days. Cells were stained with Calcein-AM (ThermoFisher, C3100MP) and ethidium homodimer (Sigma, 46043-1MG-F) in a live/dead assay: Samples were imaged with a Zeiss Axiovert microscope; 10X images were used to count live cells (green) and dead cells (red) using Image J software. Each cell type was tested with 2 biological replicates of 4 technical replicates each.

#### MTS Assay

HUVECs (8.0 × 10^3^ cells/cm^2^) and INS-1 832/13 cells (25 × 10^3^ cells/cm^2^) were plated onto flat-bottom 96-well tissue culture plates with either no coating, collagen I coating (Corning, 354249) or hP-HG coating, and grown in their respective medium. On days 1, 2, 3 and 4 after plating, CellTiter-96 (Promega, G3582) reagent was added to the cultures for 3 hours, media absorbance at 490 nm was measured (FlexStation 3, Molecular Devices). Culture media were tested in triplicate for each condition and time point. A growth curve was generated for each cell line and condition tested.

#### GSIS Assay

INS-1 832/13 cells were plated at a density of 0.5 × 10^6^ cells per well in a 24-well plate (N = 4) (Corning 3527), with either no coating, Col1 coating or hP-HG coating. Cells were expanded for 48 hours in standard medium, and grown until 95% confluent. On the day of the assay each well of cells was washed with Krebs buffer (25 mM HEPES, 115 mM NaCl, 24 mM NaHCO_3_, 5 mM KCl, 1 mM MgCl_2_, 2.5 mM CaCl_2_, 1% BSA) and pre-incubated in a very low glucose Krebs solution (0.5 mM) for 2 hours at 37 °C and 5% CO_2_. A GSIS was performed using serial low glucose (2.8 mM) and high glucose (28 mM) Krebs solutions with one hour incubations (37 °C, 5% CO_2_) for each treatment. At the end of each incubation, supernatant was collected for human C-Peptide content measurement using the Mercodia Ultrasensitive C-Peptide ELISA kit (10-1141-01).

#### Doxycycline Induction Assay

Undifferentiated H9 cells with a Doxycycline-inducible GFP cassette were passaged with Versene (Life Technologies, 15040066) into small cell clusters, pelleted and mixed into 250 µl of cold 6.0 mg/ml hydrogel. Gel and cells were pipetted into a 12-well Transwell® (Corning 3460), covering the membrane, and incubated at 37 °C for 30 minutes. After 30 minutes, 0.5 mL E8 medium was added over the gels, and 1.0 ml of medium was added to the bottom of the Transwell® compartment. Media was replaced daily for 7 days with E8 media + doxycycline (1.5 µg/ml). GFP was imaged in live cells each day with a Zeiss Axiovert 200 M microscope, and gels were marked with a nick to identify the same spot in each gel every day. On day 7, gels were fixed in 4% PFA for 30 minutes, processed for paraffin embedding, sectioning and staining.

#### Stem cell differentiation

H1 cells were differentiated toward a pancreatic endocrine fate using a protocol based on Xu *et al*.^70^, Rezania *et al*.^71^, and Pagliuca *et al*.^72^. After 11 days of differentiation, pancreatic progenitor cells were embedded in hP-HG and cultured for an additional 9 days to test for survival, apoptosis and retention of pancreatic progenitor fate. After 28 days of differentiation, islet-like clusters (ILCs) were embedded in hP-HG for an additional 4 days in end-stage medium, to test for survival, apoptosis and retention of mature beta cell marker proteins. Constructs were fixed in 4% PFA and processed for paraffin embedding and analysis.

### *In vivo* implantation

#### Transplantation of ECM into Humanized Mice

Research involving mice was performed in accordance with a protocol that was approved by the University of Wisconsin School of Medicine and Public Health Animal Care and Use Committee, and in accordance with a protocol approved by the University of Wisconsin Institutional Review Board. For immunogenicity studies, 500 µL of hP-HG was injected into the dorsal subcutaneous space of humanized mice (n = 2) (See Supplementary Methods). Additionally, human foetal pancreas (HFP) fragments from a donor allogeneic to the donor of the mouse’s human immune system was also transplanted subcutaneously into the same animal. The three grafts remained *in vivo* for a period of four weeks before the animals were sacrificed, and grafts collected for processing and immunohistochemistry.

#### ECM digestion and proteomics analysis

Protein extraction and DiLeu labelling were performed as previously described^73,74^. Briefly, same amount of protein was digested with trypsin, labelled with 12plex DiLeu reagents and fractionated by off-line strong cation exchange (SCX) chromatography. Ten fractions were collected to reduce the sample complexity. Peptides were online separated by Dionex UltiMate 3000 LC system before entering the Orbitrap Fusion Lumos tribrid mass spectrometer (San Jose, CA). The descriptions of MS settings have been previously described^73^. (More details can be found in Supplementary Methods).

Quantification was performed using an in-house software called DiLeu tool. For each protein in the native (non-decelled) pancreas, the intensity (I) was normalized using the ratio of Col1A1 intensities of the native and decelled samples, using Equation 2. The normalized native intensity can then be compared to the corresponding experimental intensity for the same protein in the decelled sample. The average intensity for each protein in each group (decelled and native) was calculated using 5 donor pancreata.

2 $${{\rm{I}}}_{norm}(Native,\,Protein\,X)={{\rm{I}}}_{exp}(Native,\,Protein\,X)\,\ast \,\frac{{{\rm{I}}}_{exp}(Decell,\,Col1A1)}{{{\rm{I}}}_{exp}(Native,\,Col1A1)}$$

#### Statistical analysis

Statistical analyses were calculated with Prism 6 for Windows (GraphPad Software, Inc.). All results are reported as mean values across biological replicates ± the standard deviation of the mean. Statistical comparisons for all samples were made using repeated measures ANOVA testing followed by Tukey’s multiple comparison test to determine significance between individual means using SAS version 9.2 (SAS Institute Inc., Cary, NC). A p-value of less than 0.05 was considered significant, and Prism’s recommended classification for significance was followed (p < 0.0001 = extremely significant (****), 0.0001 < p < 0.001 = extremely significant (***), 0.001 < p < 0.01 = very significant (**), and 0.01 < p < 0.05 = significant (*)).

### Data availability

The raw/processed data required to reproduce these findings cannot be shared at this time as the data also forms part of an ongoing study.

## Electronic supplementary material

Supplementary Information
