# Supplementary material for: Extracellular matrix scaffold and hydrogel derived from decellularized and delipidized human pancreas
Source: Sci Rep. 2018 Jul 11;8:10452. doi: 10.1038/s41598-018-28857-1 (PMC6041318; doi:10.1038/s41598-018-28857-1)
Supplement: Supplementary file 1 — Supplementary Information [file 41598_2018_28857_MOESM1_ESM.pdf]

Supplementary Information

Extracellular matrix scaffold and hydrogel derived from decellularized and delipidized human pancreas

Sara Dutton Sackett*#[1], Daniel M. Tremmel#[1], Fengfei Ma[2], Austin K. Feeney[1], Rachel M. Maguire[1], Matthew E. Brown[1], Ying Zhou[1], Xiang Li[1], Cori O'Brien[1], Lingjun Li[2,3], William J. Burlingham[1] and Jon S. Odorico[1]

# Sara Dutton Sackett and Daniel M. Tremmel contributed equally in this article

[1]Division of Transplantation, Department of Surgery, University of Wisconsin-Madison School of Medicine and Public Health, Madison, Wisconsin, USA
[2]School of Pharmacy, University of Wisconsin, Madison, Wisconsin, USA
[3]Department of Chemistry, University of Wisconsin, Madison, Wisconsin, USA

ID	Sex	Patient Age	Weight (kg)	Height (cm)	BMI	Donor Type	Blood Type	DCD/DBD	CIT (hours)	HbA1c (4.3-6.5)	Lipase (75-390)	Amylase (20-100)
8	Male	13 years	100	177.8	31.63	Head Trauma	O	DCD	7	5.9	196	190
10	Female	53 years	102	170.18	35.22	Anoxia	A	DCD	6	nd	153	35
11	Male	57 years	81.8	177.8	25.88	Cerebrovascular/Stroke	O	DBD	3	5.7	13	37
12	Male	27 years	30	121.92	20.18	Other Specify	O	DCD	20.5	5.8	15	100
13	Male	39 years	97.7	170.18	33.73	Cerebrovascular/Stroke	O	DBD	15.5	nd	390	71
14	Male	55 years	86	177.8	27.2	Head Trauma	A	DBD	8	4.8	12	40
15	Male	31 years	95	182.88	28.4	Head Trauma	A	DBD	9	5	22	43
16	Male	53 years	80	172.72	26.82	Anoxia	A	DBD	19	nd	14	316
23	Male	58 years	81.7	172.72	27.39	Cerebrovascular/Stroke	A	DBD	6.5	nd	19	35
27	Female	45 years	92.8	172.72	31.11	Cerebrovascular/Stroke	A	DBD	13.5	nd	9	55
35	Female	48 years	53	160.02	20.7	Head Trauma	O	DBD	5	5.4	19	31

Table S1: Characteristics of human cadaver donors providing pancreata for decellularization.
Abbreviations: donor after cardiac death (DCD); donor after brain death (DBD); cold ischemic time (CIT); hemoglobin a1C (HbA1c); Body mass index (BMI).

Antibody	Product Number	Dilution	Manufacturer
Mouse anti-Collagen I	ab88147	1:250	Abcam
Rabbit anti-Pdx1	ab134150	1:1000	Abcam
Mouse anti-Insulin	I2018	1:10000	Sigma-Aldrich
Rabbit anti-Glucagon	ab92517	1:2000	Abcam
Rabbit anti-Ki67	ab16667 clone SP6	1:500	Abcam
Rabbit anti-Caspase3	9662	1:500	Cell Signalling
Mouse anti-Collagen IV	ab6586	1:1000	Abcam
Rabbit anti-Laminin	L9393	1:200	Sigma-Aldrich
Mouse anti-HLA ABC	ab70328	1:100	Abcam
Mouse anti-HLA DR	ab80658	1:1000	Abcam
Mouse anti-Nkx6.1	F55A12-s	1:100	DSHB
Rabbit anti-vWF	A008202-5	1:500	Dako
Mouse anti-VE Cadherin	sc-9989	1:100	Santa Cruz Biotechnology
Anti-human CD3	ab134093	1:500	Abcam
Anti-human CD8	ab108343	1:100	Abcam
Anti-human CD20	555677	1:500	BD Pharmingen
Anti-human CD45	555491	1:20	BD Pharmingen
Anti-human CD68	M0814	1:1000	Dako
Anti-mouse CD68	ab31630	1:100	Abcam
Anti-human FoxP3	ab10563	1:300	Abcam

Table S2: Antibodies for immunocytochemistry and immunohistochemistry.

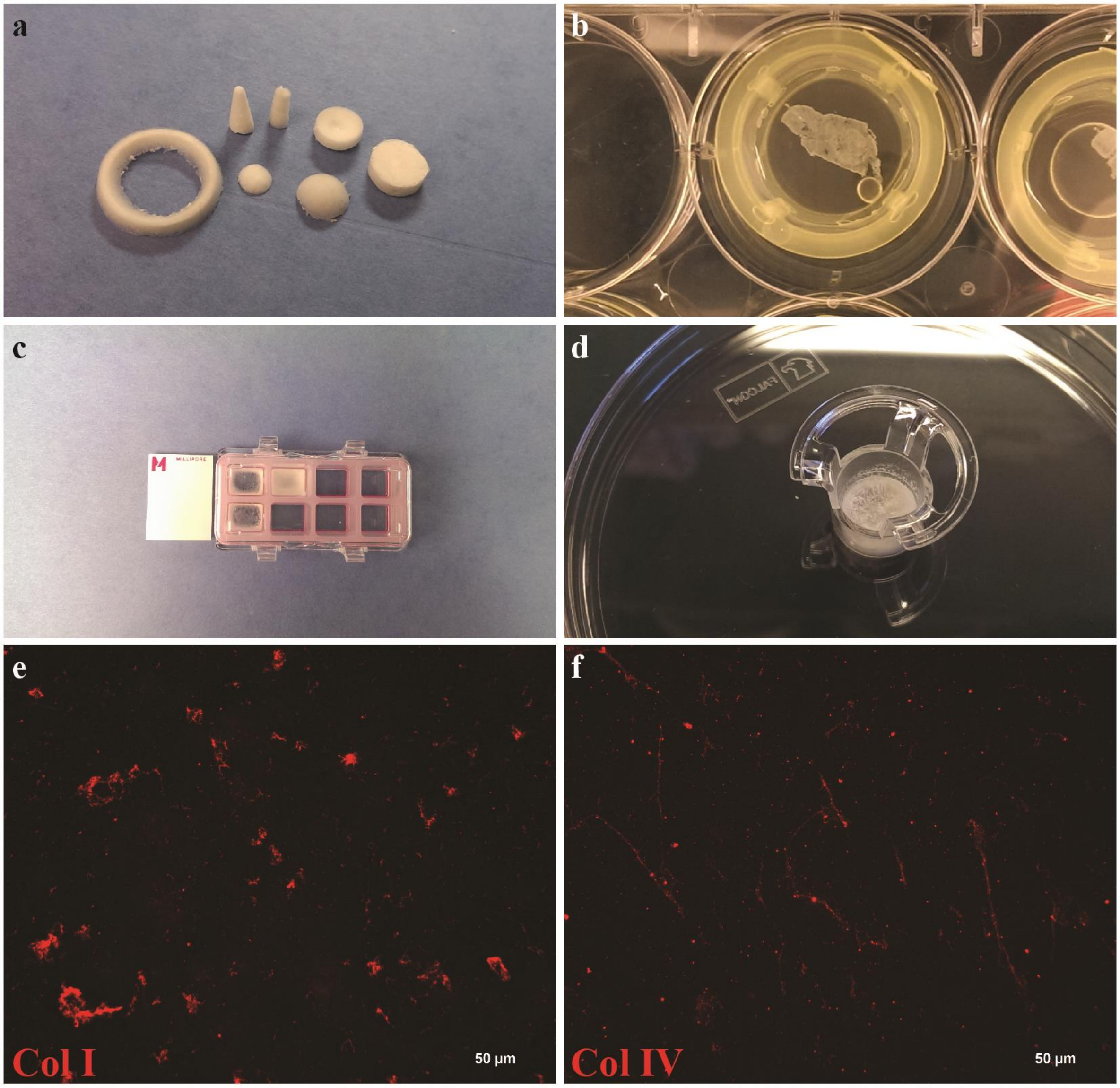

Fig. S1: Applications for decellularized pancreas ECM. hP-ECM and hP-HG are able to be manipulated to produce several types of acellular natural matrix constructs, including intact 3D matrix (**a**), cut in thin slices (**b**), placed in tissue culture vessels such as chamber slides (**c**) Transwells (**d**) and applied in thin coatings to tissue culture plate show in **e** and **f** stained with collagen I and IV.

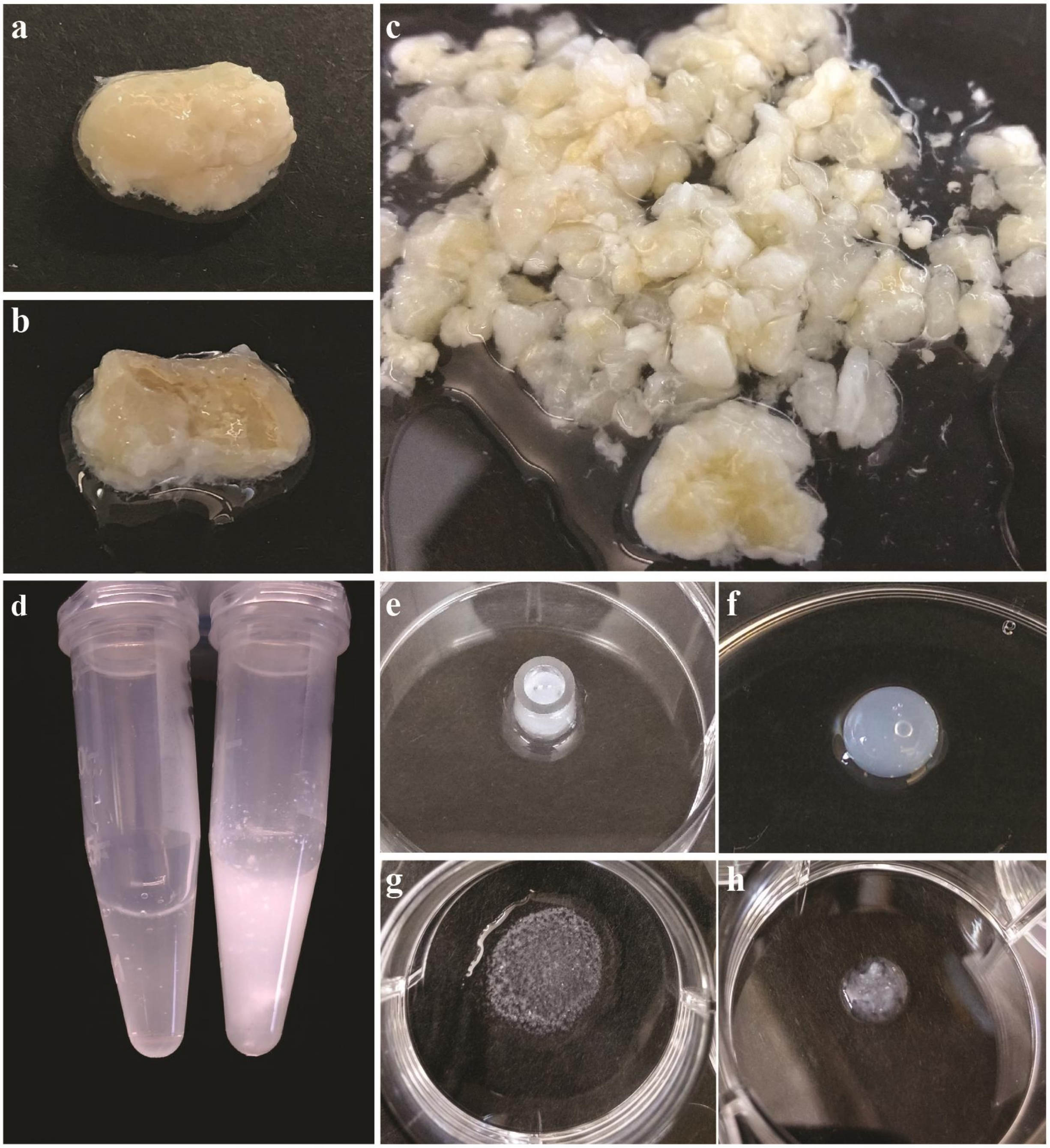

Fig. S2: High fat content inhibits solubilization and gelation. **a**) Cube of spin-decellularized pancreas, **b**) bisected to reveal a yellow interior with higher fat content. **c**) Several cut up pieces with yellow interiors. Following pepsin digestion, the pre-gel solution is cloudier following spin-decell (**d**, right) compared to homogenized decell (**d**, left). When neutralized and gelled in a mold (**e**) the homogenized-decell consistently yields a sturdy gel (**f**) while the spin-decell results in no gelation (**g**) or incomplete gelation (**h**) - depending on the initial fat content of the donor organ.

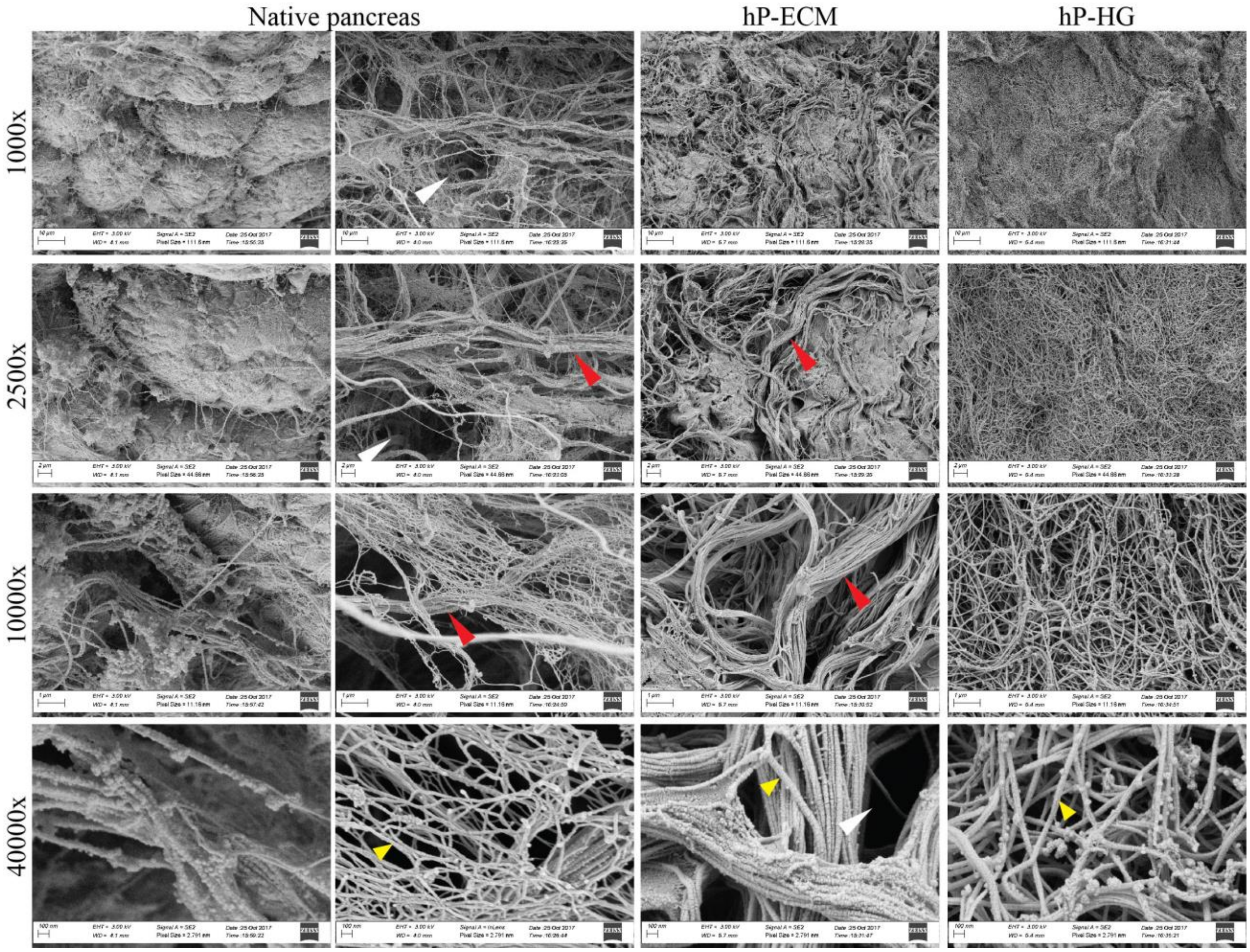

Fig S3: Organisation of native pancreas and decellularized hP-ECM and hP-HG by SEM. Native pancreas demonstrating cells organized in dense clusters and covered by connective tissues of high and low densities. Long white arrows indicate empty space within the ECM, potentially suitable for cell occupation. Long red arrows indicate collagen fibril bundle structures, retained within the hP-ECM. Short yellow arrows indicate collagen fibrils, present in the native tissue, the hP-ECM as well as the hP-HG.

	Protein Group	Type	Gene	Name	Intensity
1	Core matrisome	Collagens	COL1A2	Collagen Type 1 Alpha 1 Chain	1.97E+09
2	Core matrisome	Collagens	COL1A1	Collagen Type 1 Alpha 2 Chain	5.17E+09
3	Core matrisome	Collagens	COL5A2	Collagen Type 5 Alpha 2 Chain	1.53E+08
4	Core matrisome	Collagens	COL5A1	Collagen Type 5 Alpha 1 Chain	1.24E+08
5	Core matrisome	Collagens	COL3A1	Collagen Type 3 Alpha 1 Chain Isoform 1	1.42E+09
6	Core matrisome	Collagens	COL3A1	Collagen Type 3 Alpha 1 Chain Isoform 2	4.00E+06
7	Core matrisome	Collagens	COL6A1	Collagen Type 6 Alpha 1 Chain	6.32E+07
8	Core matrisome	Collagens	COL6A3	Collagen Type 6 Alpha 3 Chain	7.46E+07
9	Core matrisome	Collagens	COL6A2	Collagen Type 6 Alpha 2 Chain Isoform 2C2	1.83E+06
10	Core matrisome	Collagens	COL6A2	Collagen Type 6 Alpha 2 Chain Isoform 2C2A	6.23E+04
11	Core matrisome	Collagens	COL4A2	Collagen Type 4 Alpha 2 Chain	6.38E+07
12	Core matrisome	Collagens	COL14A1	Collagen Type 14 Alpha 1 Chain	2.81E+06
13	Core matrisome	Collagens	COL5A3	Collagen Type 5 Alpha 3 Chain	3.99E+07
14	Core matrisome	Collagens	COL2A1	Collagen Type 2 Alpha 1 Chain	2.81E+07
15	Core matrisome	Collagens	COL16A1	Collagen Type 16 Alpha 1 Chain	1.22E+07
16	Core matrisome	Collagens	COL4A4	Collagen Type 4 Alpha 4 Chain	8.56E+05
17	Core matrisome	Collagens	COL4A1	Collagen Type 4 Alpha 1 Chain	2.90E+07
18	Core matrisome	Collagens	COL18A1	Collagen Type 18 Alpha 1 Chain	1.66E+06
19	Core matrisome	Collagens	COL11A1	Collagen Type 11 Alpha 1 Chain	6.30E+05
20	Core matrisome	Collagens	COL15A1	Collagen Type 15 Alpha 1 Chain	1.26E+06
21	Core matrisome	Collagens	COL8A1	Collagen Type 8 Alpha 1 Chain	7.11E+06
22	Core matrisome	Collagens	COL6A6	Collagen Type 6 Alpha 6 Chain	1.03E+06
23	Core matrisome	Collagens	COL28A1	Collagen Type 28 Alpha 1 Chain	2.34E+06
24	Core matrisome	Collagens	COL4A5	Collagen Type 4 Alpha 5 Chain	1.42E+05
25	Core matrisome	Collagens	COL4A3	Collagen Type 4 Alpha 1 Chain	3.60E+05
26	Core matrisome	Collagens	COL21A1	Collagen Type 21 Alpha 1 Chain	4.20E+06
27	Core matrisome	Collagens	COL11A2	Collagen Type 11 Alpha 2 Chain	3.69E+05
28	Core matrisome	Collagens	COL12A1	Collagen Type 12 Alpha 1 Chain	7.56E+05
29	Core matrisome	Collagens	COL10A1	Collagen Type 10 Alpha 1 Chain	4.20E+05
30	Core matrisome	Collagens	COL13A1	Collagen Type 13 Alpha 1 Chain	5.28E+05
31	Core matrisome	Collagens	COL7A1	Collagen Type 7 Alpha 1 Chain	1.01E+06
32	Core matrisome	Collagens	COL9A3	Collagen Type 9 Alpha 3 Chain	6.77E+05
33	Core matrisome	Collagens	COL8A2	Collagen Type 8 Alpha 2 Chain	4.86E+05
34	Core matrisome	ECM Glycoproteins	LAMA5	Laminin Subunit Alpha 5	1.61E+06
35	Core matrisome	ECM Glycoproteins	LAMC1	Laminin Subunit Gamma 1	3.31E+06
36	Core matrisome	ECM Glycoproteins	LAMB1	Laminin Subunit Beta 1	1.93E+06
37	Core matrisome	ECM Glycoproteins	FBN1	Fibrillin 1	3.35E+06
38	Core matrisome	ECM Glycoproteins	LAMA2	Laminin Subunit Alpha 2	2.20E+06
39	Core matrisome	ECM Glycoproteins	LAMB2	Laminin Subunit Beta 2	9.19E+05
40	Core matrisome	ECM Glycoproteins	AGRN	Agrin	1.10E+06
41	Core matrisome	ECM Glycoproteins	NID2	Nidogen 2	9.52E+05
42	Core matrisome	ECM Glycoproteins	NID1	Nidogen 1	6.23E+05
43	Core matrisome	ECM Glycoproteins	FGA	Fibrinogen Alpha Chain	6.99E+05
44	Core matrisome	ECM Glycoproteins	FN1	Fibronectin 1	7.34E+05
45	Core matrisome	ECM Glycoproteins	TINAGL1	Tubulointerstitial Nephritis Antigen Like 1	4.51E+05
46	Core matrisome	ECM Glycoproteins	FRAS1	Fraser Extracellular Matrix Complex Subunit 1	1.49E+05
47	Core matrisome	ECM Glycoproteins	FGB	Fibrinogen Beta Chain	2.96E+05
48	Core matrisome	ECM Glycoproteins	FGG	Fibrinogen Gamma Chain	3.31E+05
49	Core matrisome	ECM Glycoproteins	VTN	Vitronectin	2.98E+05
50	Core matrisome	ECM Glycoproteins	LAMA4	Laminin Subunit Alpha 4	3.71E+05
51	Core matrisome	ECM Glycoproteins	MFGE8	Milk Fat Globule-EGF Factor 8 Protein	1.98E+05
52	Core matrisome	ECM Glycoproteins	DPT	Dermatopontin	1.21E+06
53	Core matrisome	ECM Glycoproteins	EMILIN1	Elastin Microfibril Interfacer 1	1.54E+05
54	Core matrisome	ECM Glycoproteins	LAMC3	Laminin Subunit Gamma 3	2.81E+05
55	Core matrisome	ECM Glycoproteins	TNXB	Tenascin XB	1.44E+05
56	Core matrisome	ECM Glycoproteins	POSTN	Periostin	8.03E+04
57	Core matrisome	ECM Glycoproteins	TGFBI	Transforming Growth Factor Beta Induced	1.24E+05
58	Core matrisome	ECM Glycoproteins	IGFBP7	Insulin Like Growth Factor Binding Protein 7	1.86E+05
59	Core matrisome	ECM Glycoproteins	VWA5A	Von Willebrand Factor A Domain Containing 5A	5.47E+04
60	Core matrisome	ECM Glycoproteins	THBS1	Thrombospondin 1	2.95E+04
61	Core matrisome	ECM Glycoproteins	NPNT	Nephronectin	3.68E+04
62	Core matrisome	ECM Glycoproteins	FBLN1	Fibulin 1	2.75E+04
63	Core matrisome	ECM Glycoproteins	IGFBP2	Insulin Like Growth Factor Binding Protein 2	2.38E+04
64	Core matrisome	ECM Glycoproteins	LTBP1	Latent Transforming Growth Factor Beta Binding Protein	1.02E+05
65	Core matrisome	Proteoglycans	HSPG2	Perlecan	6.59E+06
66	Core matrisome	Proteoglycans	BGN	Biglycan	1.06E+07
67	Core matrisome	Proteoglycans	PRELP	Prolargin	6.93E+06
68	Core matrisome	Proteoglycans	DCN	Decorin	1.16E+07
69	Core matrisome	Proteoglycans	ASPN	Asporin	4.94E+06
70	Core matrisome	Proteoglycans	LUM	Lumican	2.76E+06
71	Core matrisome	Proteoglycans	OGN	Osteoglycin	7.76E+05
72	Core matrisome	Proteoglycans	FMOD	Fibromodulin	3.16E+04
73	Matrisome-associated	ECM Regulators	PRSS1	Protease, Serine 1	1.14E+06
74	Matrisome-associated	ECM Regulators	PRSS3	Protease, Serine 3	6.74E+05
75	Matrisome-associated	ECM Regulators	CELA3A	Chymotrypsin Like Elastase Family Member 3A	7.89E+05
76	Matrisome-associated	ECM Regulators	PRSS2	Protease, Serine 2	3.99E+05
77	Matrisome-associated	ECM Regulators	CELA2A	Chymotrypsin Like Elastase Family Member 2A	8.69E+05
78	Matrisome-associated	ECM Regulators	TGM2	Transglutaminase 2	9.74E+05
79	Matrisome-associated	ECM Regulators	CELA3B	Chymotrypsin Like Elastase Family Member 3B	2.78E+05
80	Matrisome-associated	ECM Regulators	CTSD	Cathepsin D	7.98E+05
81	Matrisome-associated	ECM Regulators	CELA2B	Chymotrypsin Like Elastase Family Member 2B	1.52E+05
82	Matrisome-associated	ECM Regulators	AMBP	Alpha-1-Microglobulin/Bikunin Precursor	6.31E+05
83	Matrisome-associated	ECM Regulators	SERPINB1	Serpin Family B Member 1	6.24E+05
84	Matrisome-associated	ECM Regulators	SERPINA3	Serpin Family A Member 3	9.15E+04
85	Matrisome-associated	ECM Regulators	SERPINA1	Serpin Family A Member 1	3.81E+05
86	Matrisome-associated	ECM Regulators	SERPINI2	Serpin Family I Member 2	6.19E+05
87	Matrisome-associated	ECM Regulators	CSTB	Cystatin B	1.25E+05
88	Matrisome-associated	ECM Regulators	CTSB	Cathepsin B	3.34E+05
89	Matrisome-associated	ECM Regulators	SERPINH1	Serpin Family H Member 1	1.05E+04
90	Matrisome-associated	ECM Regulators	A2M	Alpha-2-Macroglobulin	3.57E+04
91	Matrisome-associated	ECM Regulators	ITIH5	Inter-α-Trypsin Inhibitor Heavy Chain Family Member 5	5.27E+04
92	Matrisome-associated	ECM Regulators	CST3	Cystatin C	9.04E+04
93	Matrisome-associated	ECM Regulators	SERPING1	Serpin Family G Member 1	1.67E+04
94	Matrisome-associated	ECM Regulators	KNG1	Kininogen 1	2.71E+04
95	Matrisome-associated	ECM-affiliated Proteins	ANXA4	Annexin A4	7.93E+05
96	Matrisome-associated	ECM-affiliated Proteins	ANXA6	Annexin A6	4.42E+05
97	Matrisome-associated	ECM-affiliated Proteins	ANXA2	Annexin A2	6.07E+05
98	Matrisome-associated	ECM-affiliated Proteins	REG1A	Regenerating Family Member 1 Alpha	1.11E+06
99	Matrisome-associated	ECM-affiliated Proteins	ANXA1	Annexin A1	2.75E+05
100	Matrisome-associated	ECM-affiliated Proteins	REG1B	Regenerating Family Member 1 Beta	1.79E+05
101	Matrisome-associated	ECM-affiliated Proteins	LMAN1	Lectin, Mannose Binding 1	3.77E+05
102	Matrisome-associated	ECM-affiliated Proteins	LGALS4	Galectin 4	4.07E+05
103	Matrisome-associated	ECM-affiliated Proteins	LGALS1	Galectin 1	8.23E+05
104	Matrisome-associated	ECM-affiliated Proteins	ANXA11	Annexin A11	2.71E+05
105	Matrisome-associated	ECM-affiliated Proteins	ANXA3	Annexin A3	9.49E+04
106	Matrisome-associated	ECM-affiliated Proteins	LGALS2	Galectin 2	1.76E+05
107	Matrisome-associated	ECM-affiliated Proteins	ANXA5	Annexin A5	1.08E+04
108	Matrisome-associated	ECM-affiliated Proteins	ANXA7	Annexin A7	1.63E+04
109	Matrisome-associated	ECM-affiliated Proteins	LGALS8	Galectin 8	3.44E+04
110	Matrisome-associated	ECM-affiliated Proteins	LGALS3	Galectin 3	6.71E+03
111	Matrisome-associated	ECM-affiliated Proteins	REG3A	Regenerating Family Member 3 Alpha	7.89E+04
112	Matrisome-associated	ECM-affiliated Proteins	C1QTNF2	C1q And TNF Related 2	6.60E+04
113	Matrisome-associated	ECM-affiliated Proteins	C1QTNF5	C1q And TNF Related 5	2.33E+05
114	Matrisome-associated	ECM-affiliated Proteins	HPX	Hemopexin	4.07E+04
115	Matrisome-associated	Secreted Factors	S100A13	S100 Calcium Binding Protein A13	8.68E+04
116	Matrisome-associated	Secreted Factors	HRNR	Hornerin	1.79E+04
117	Matrisome-associated	Secreted Factors	S100A10	S100 Calcium Binding Protein A10	2.41E+04
118	Matrisome-associated	Secreted Factors	S100A11	S100 Calcium Binding Protein A11	8.73E+03
119	Matrisome-associated	Secreted Factors	CFC1B	CFC1B	2.33E+04
120	Matrisome-associated	Secreted Factors	HCFC1	Host Cell Factor C1	5.72E+04

Fig. S4: Full list of all 120 matrisome and matrisome-associated proteins identified in the decellularized hP-ECM. Proteins were classified using the MatrixDB database.

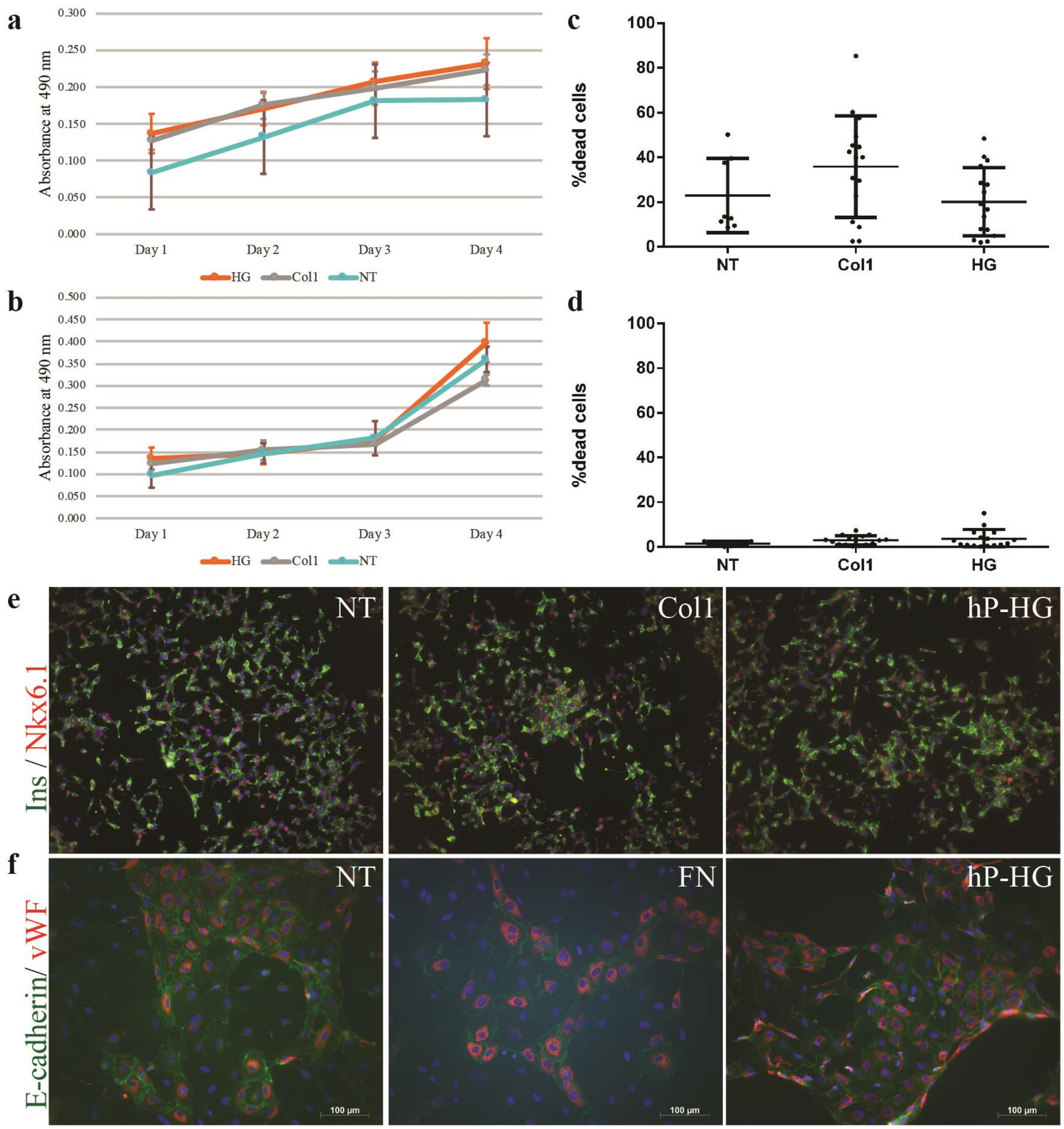

Fig. S5: *In vitro* cytocompatibility evaluation of hP-HG using HUVECs and INS-1 832/13 cells. Cells were cultured for 4 days on either tissue culture plastic (NT, not treated), collagen I (Col 1), or human pancreatic ECM hydrogel (hP-HG). Cells were analysed daily using the MTS assay, at the end of the culture period by live:dead staining protocol and by specific marker staining. **a**) HUVEC MTS growth curve. **b**) INS-1 832/13 cells MTS growth curve. **c** and **d**) % dead cells quantified at day 4 of culture for HUVECs and INS-1 832/13 cells, respectively. **e**) Insulin (green) and Nkx6.1 (red) staining of INS-1 832/13 cells cultured under the 3 different conditions at day 4 of culture. **f**) E-cadherin (green) and von Willebrands Factor (vWF, red) staining of HUVECS cultured under the 3 different conditions at day 4 of culture.

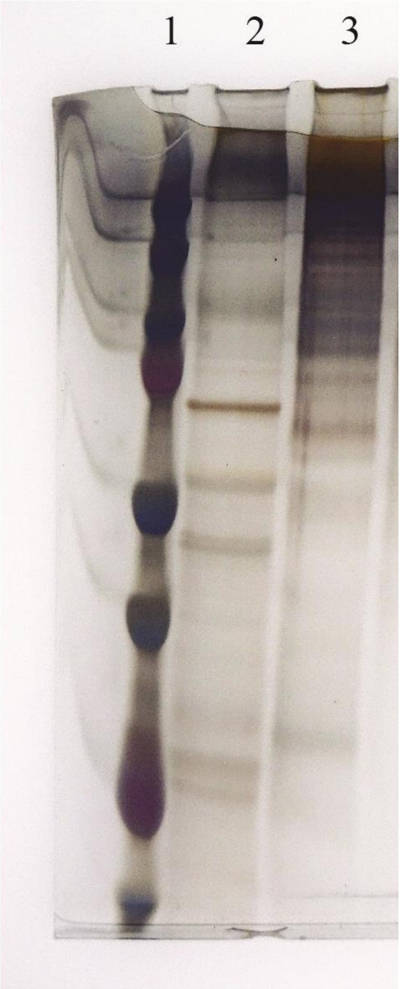

Fig. S6: Full length image of the SDS-PAGE gel shown in Figure 6. Lane 1 is loaded with the Precision Plus Protein Dual Color Standard. Lane 2 is loaded with 10 µg of the native pancreas from the same donor as Lane 3, loaded with 10 µg of decellularized pancreatic ECM. The remaining lanes of the gel contained samples unrelated to this project. The image shown in Figure 6a was cropped slightly to remove the edges of the gel, and rotated slightly to straighten the lanes.

Supplementary Methods.

Pancreata Procurement.

Pancreata are deemed unsuitable for transplantation for some of the following reasons: vascular damage or anomaly, high BMI, or duodenal injury or anomaly after visual inspection. Organs were selected from donors with no history or evidence of pancreatitis, diabetes, pancreatic cancer, hepatitis or HIV infection and were from donation after circulatory death (DCD) or donation after brain death (DBD). Organs were recovered after *in situ* flush with UW Solution [ViaSpan® (TEVA Pharmaceuticals USA, Inc., North Wales, Pennsylvania), SPS-1, (Organ Recovery Systems, Inc. Itasca, Illinois), or UW Belzer® Cold Storage Solution (Bridge to Life, Ltd. Columbia, South Carolina)], and stored in the same solution at 4˚C until tissues were processed within 24 hours of recovery. No organs/tissue were procured from prisoners.

Modified Folch Method

30 mg of lyophilized ECM was weighed; 2 ml of 1:3 (v:v) chloroform:methanol solution was added to each sample. Samples were incubated for 30 minutes shaking at RT, centrifuged (4300 rpm, 5 min), and the supernatant was decanted into a new tube. 800 µl of water and 2.5 ml of chloroform was added to the supernatant, and tubes were vigorously shaken. Samples were centrifuged (2200 rpm, 5 min) and the upper phase was removed. The lower phase was allowed to air dry in a fume hood for 4-7 days until dry; the resulting lipid material was weighed to determine lipid content of the original sample.

Hydrogel Formation.

Pepsin was used at a ratio of 0.1 mg pepsin per 1 mg ECM and solubilized in 0.01 M HCl prior to being added to the ECM. The digestion was prepared such that the final gel would have a density of 8 mg ECM/ml gel. This required a volume of 818 µl of 0.01 M HCl per 1 mg of lyophilized ECM. ECM was digested for 72-96 hours at RT until the solution was transparent and free of undigested pieces. If a significant amount of undigested matrix was still present after 48 hours of digestion, the solution was homogenized and digested for another 48 hours at RT. Neutralization of the acidic digest solution was achieved by mixing ice cold 0.1 M NaOH (equal to 1/10 the volume of acidic digest) and 10X PBS (equal to 1/9 the total volume of digest + NaOH used) and adding this mixed solution to the digested ECM at 4˚C. Mixed pre-gel solution is then placed at 37˚C for 30 minutes for gelation to occur. If not used immediately, the gel was lyophilized and stored at -80˚C.

Generation of Humanized Mice.

Research involving mice was performed in accordance with a protocol that was approved by the University of Wisconsin School of Medicine and Public Health Animal Care and Use Committee, and in accordance with a protocol approved by the University of Wisconsin Institutional Review Board. Humanized mice were generated similarly to previously published reports [65-67]. Briefly, NOD-*scid* *IL2rγ*^{null} (NSG) mice aged 7–8 weeks, obtained from Jackson Laboratories (stock #005557), were conditioned with sublethal total body irradiation with 250 RAD via an X-RAD 320ix irradiator (Precision X-Ray, North Branford, CT) and within 4 hours transplanted with 1mm^3 human foetal thymus fragments (14-20 weeks gestation and obtained from Advanced Bioscience Resource (Alameda, CA)) into the kidney subcapsular space. Immediately following surgery, mice were intravenously injected with 40,000 purified CD34+ cells, which were isolated from autologous liver tissue. The CD34+ cells were isolated via magnetically activated cell sorter (MACS) separation system (Militenyi Biotec, Auburn, CA). Mice were monitored for engraftment of human white blood cells (hCD45+) at 8 and 12 weeks and are considered

successfully humanized with presence of greater than 25% hCD45+ cells. Further details are available in supplementary materials.

Proteomics analysis.

The OMSSA proteomic Analysis Software Suite (COMPASS) was used for peptide identification and searched against Homo sapiens Uniprot database. Trypsin was selected as the enzyme and maximum of two missed cleavages were allowed. Precursor and fragment ion tolerance was set to 25 ppm and 0.02 Da. DiLeu labelling on N-termini, lysine residue (+145.1267748 Da), and carbamidomethylation of cysteine residues (+57.02146 Da) were chosen as static modifications. Variable modifications included methionine oxidation (+15.99492 Da) and DiLeu labeling on tyrosine residue (+145.1267748). Results were filtered at 1% false discovery rate (FDR) for both peptide and protein.
